# The Intertwined Victimhood Narrative and the Challenge to Chinese Men’s Gender Role Legitimacy

**DOI:** 10.3390/bs16091609

**Published:** 2026-09-09

**Authors:** Jiaying Liu, Yue Zhang, Huiyue Shi, Zhaoming Chen, Lian Xue, Yan Xu

**Affiliations:** 1 Beijing Key Laboratory of Applied Experimental Psychology, National Demonstration Center for Experimental Psychology Education (Beijing Normal University), Faculty of Psychology, Beijing Normal University, Beijing 100875, China; 202231061023@mail.bnu.edu.cn (J.L.);; 2 School of Psychology, South China Normal University, Guangzhou 510631, China; sherryz1014@gmail.com; 3 Department of Educational Psychology, University of Utah, Salt Lake City, UT 84112, USA; zhaoming.chen@utah.edu; 4 School of Psychology, The University of Sydney, Sydney, NSW 2006, Australia; lian.xue@sydney.edu.au

**Keywords:** gender equality, the Intertwined Victimhood Narrative, discrimination against men, gender role, legitimacy of gender roles

## Abstract

Gender inequality is often framed in terms of women’s disadvantage, whereas men’s gender-role constraints are overlooked or discussed in opposition to gender equality. We introduce the Intertwined Victimhood Narrative (IVN), which frames men’s and women’s constraints as interconnected consequences of a shared gender-role system. Two experiments recruited Chinese men through Credamo and randomly assigned them to narrative conditions. Study 1 (*N* = 200) compared the IVN with a Women-Centric Victimhood Narrative (WCVN) to test whether it would increase men’s recognition of constraints affecting themselves and weaken acceptance of existing gender roles. The IVN increased perceived discrimination against men without reducing perceived discrimination against women, and reduced general gender-role legitimacy. Study 2 (*N* = 270) further tested whether the IVN would reduce prescriptive gender stereotypes concerning both men and women. Men exposed to the IVN endorsed these stereotypes less strongly overall than those in the WCVN and control conditions, and none of the interactions involving the narrative condition reached significance. The WCVN did not differ from the control condition. These findings suggest that interconnected framing may broaden men’s stake in questioning restrictive gender arrangements and that the consequences of discussing male victimhood depend on its framing.

## 1. Introduction

Because women face more severe and salient forms of inequality, gender inequality is often defined and described primarily from women’s perspective (e.g., Radke et al., 2020). These narratives help women make sense of their experiences, politicize personal hardship, and build alliances with other women through shared recognition of discrimination (Hanisch, 1969). Yet men are also constrained by gender inequality, although their suffering is seldom incorporated into dominant accounts of the problem. Rigid gender roles restrict men’s emotional expression, stigmatize dependency and vulnerability, pressure them to serve as providers, and devalue their participation in caregiving and other traditionally feminine domains (Bem & Lewis, 1975; O’Neil, 1981). Men who fail to meet these expectations may face shame, social devaluation, and strain in both work and family life. Importantly, acknowledging men’s constraints under patriarchy does not imply that men and women are disadvantaged in equivalent ways or to equivalent degrees. Patriarchal arrangements continue to privilege men as a group relative to women in access to power, status, and resources, even as they impose restrictive role expectations on men.

When the costs of gender inequality for men are absent from dominant narratives, men may fail to recognize that they, too, have a stake in gender equality. Instead, they are more often positioned as bystanders or benevolent helpers rather than as people whose own lives are also shaped by restrictive gender arrangements (e.g., Droogendyk et al., 2016; Radke et al., 2020; Subašić et al., 2008; Wiley et al., 2021). As a result, they may be more concerned with protecting the image and interests of their own group than with actively supporting gender equality. Moreover, when men’s victimhood remains unaddressed, a vacuum may be filled by adversarial narratives that frame women as the source of men’s frustrations. Such narratives have become increasingly visible in China, where economic insecurity, breadwinning and marriage pressures, and difficulties in intimate relationships may be reframed as evidence that ordinary men are exploited by women or disadvantaged by feminism (He & Fan, 2026; Huang, 2023; Wu & Zhang, 2025). Similar patterns have been documented in South Korea and the U.S., where perceived status decline and competitive victimhood are associated with reverse-discrimination beliefs and negative attitudes toward women (Kim, 2024; Lee et al., 2025; Zehnter et al., 2021). These narratives attribute men’s difficulties to women or feminism rather than to the gender system, thereby positioning male victimhood in opposition to women’s claims and gender equality.

Accordingly, the central question is not simply whether men’s constraints should be acknowledged, but how they should be framed. We introduce the Intertwined Victimhood Narrative (IVN), which situates men’s and women’s constraints within the same gender system and emphasizes that their role-based constraints are mutually linked rather than opposed. The IVN builds on emerging common-victimhood research but goes beyond acknowledging that both genders experience harm: it emphasizes the interdependence of their constraints and examines whether this framing leads men to question the legitimacy of the gender-role system itself.

The overarching goal of the present research was to examine whether the IVN could engage Chinese men more effectively than the traditional Women-Centric Victimhood Narrative (WCVN). Two research questions guided the investigation. First, would the IVN increase men’s recognition that they themselves are constrained by gender inequality? Second, would the IVN weaken men’s endorsement of the legitimacy of existing gender roles? More specifically, would this effect be limited to roles imposed on men, or would it extend to roles prescribed for women as well? We tested these questions primarily through breadwinner–caregiver expectations that are particularly salient in contemporary China, while treating the underlying logic of mutually reinforcing gender roles as potentially broader.

### 1.1. The Intertwined Victimhood Narrative

The IVN frames gender inequality as a system that constrains both men and women through differentiated yet mutually linked role expectations. Ostensibly, the gender inequalities experienced by men and women seem to belong to different domains and manifest in different ways (Eisler & Skidmore, 1987; Gillespie & Eisler, 1992; O’Neil et al., 1986; Taylor, 2020). For example, discrimination against men often manifests as restrictions on emotional expression and disdain for those who lack social resources, while discrimination against women is more commonly associated with sexual violence and objectification. However, these challenges are deeply connected, both stemming from the mutually exclusive gender roles that shape and constrain individuals’ behaviors and identities.

Although gender is more fluid and diverse than a simple binary framework implies, the present research focuses on binary gender role expectations because these remain the dominant way in which gender inequality is socially organized and narratively communicated in everyday life.

#### 1.1.1. Theoretical Foundations of the IVN

Gender roles, as cognitive schemas, distinctly delineate the behaviors, traits, and social roles deemed appropriate for men and women (Bem, 1981). These roles often exaggerate gender differences, leading to social constraints. However, empirical evidence suggests that many presumed gender differences are considerably smaller than such gender roles imply. The gender similarities hypothesis (Hyde, 2005) is supported by numerous meta-analyses. Men and women perform similarly on most psychological variables, with substantial overlap in their task performance distributions (Hyde, 2014; Zell et al., 2015). Significant gender differences, observed in a limited number of tasks, can be attributed to specific contextual factors. For example, gender differences in helping behaviors may diminish when such behaviors occur in private settings. Therefore, the binary gender roles that people follow are not natural but rather socially constructed.

Consequently, rigid gender roles restrict individuals’ ability to express their true selves and adapt to complex social contexts. Individuals who have deeply internalized gender roles are sex-typed. Their behaviors are guided by gender schemas, leading to narrow expression of either masculinity or femininity. In contrast, individuals less influenced by gender schemas display a more flexible approach to self-expression, including androgynous individuals and undifferentiated individuals. Moreover, because gender roles are overly rigid, they hinder individuals’ adaptability. Bem and Lewis (1975) found that androgynous individuals have better social adaptability than sex-typed individuals, because the latter are restricted in behaviors aligning with their internalized gender roles in different situations. For example, men experience pressure to be the primary breadwinner, encouraging men to contribute to family life primarily through financial provision rather than caregiving or emotional involvement (O’Neil, 2008). Women are also expected to be caregivers (Taylor, 2020). This expectation often conflicts with workplace requirements for independence, resulting in lower work commitment and greater difficulty advancing to leadership positions. Women may also feel guilty and anxious about prioritizing career over family.

By intertwined constraints, we mean that prescriptions imposed on one gender help sustain complementary prescriptions imposed on the other. Gender roles not only organize unequal expectations, opportunities, and responsibilities, but also define men and women in relation to one another through contrasting and complementary expectations. Because these roles are structured around gender differentiation, prescribing a particular role for one gender often implies a contrasting role for the other. For example, defining women as primary caregivers helps sustain the expectation that men should be primary providers, while defining men as providers reinforces women’s assignment to caregiving. In this sense, the constraints faced by men and women are not independent: each set of constraints is partly maintained through the complementary role prescribed for the other gender.

This interdependence helps explain why asymmetric gender-role change has become an important concern in understanding the persistence of gender inequality (Croft et al., 2015, 2021). Although gender roles and stereotypes have changed substantially in many societies, these changes have often been asymmetric for women and men. Women’s roles have expanded more rapidly into agentic and historically male-dominated domains, whereas men’s movement into communal roles, caregiving, and female-dominated occupations has generally proceeded more slowly. This asymmetry matters not only because men have gained less freedom to cross traditional gender boundaries, but also because slower expansion of men’s caregiving roles can preserve an unequal division of domestic labor and thereby limit women’s opportunities in other domains. Indeed, although men’s participation in childcare and domestic labor has increased, women continue to shoulder a disproportionate share of these responsibilities. Thus, asymmetric gender-role change reflects the intertwined nature of gendered constraints: restrictions affecting one gender can sustain restrictions affecting the other. This interdependence provides the conceptual basis for the IVN, which frames gender inequality as a system of interconnected constraints affecting both women and men.

#### 1.1.2. Previous Gender Inequality Narratives of Victimhood

Although the negative impacts of gender roles on both genders have long been acknowledged (e.g., Glick & Fiske, 1999; O’Neil, 2008), most existing research continues to focus on the WCVN, which emphasizes the inequality women face. However, a few recent studies have begun to explore more inclusive frameworks for describing gender inequality, moving beyond the traditional WCVN. Most of the work reviewed below has been conducted in Western contexts.

For example, Subašić et al. (2018) found that using inclusive terms like “parent” instead of “mother” increased men’s collective action intentions regarding flexible parental leave. Using an Italian sample, Vázquez et al. (2024) examined how different ways of framing men’s suffering shape men’s responses to gender equality. In particular, they compared a male victimhood narrative with more confrontational messages that strongly condemned men’s immorality. Relative to a control condition, the male victimhood narrative increased collective action intentions, openness to feminist perspectives, and identification with feminism among men who strongly endorsed traditional gender roles. By contrast, the more condemnatory framing did not produce similar effects. These findings suggest that acknowledging men’s suffering may be more effective than male blaming in engaging men who are strongly invested in traditional gender roles. However, this line of work still focuses primarily on whether men will support women’s causes, rather than on whether men will come to understand gender inequality as a shared system of constraints that should be questioned for both genders. The IVN may therefore offer a further step by linking men’s and women’s constraints within the same gender system, making it possible to examine not only allyship on behalf of women, but also men’s willingness to question gender-role legitimacy itself.

The Covictimization Narrative (Hardacre & Subašić, 2022), examined in American and British contexts, is most similar to the IVN, as it also highlights the gender inequalities faced by both men and women, such as men’s reduced encouragement to engage in caregiving roles and women’s continued disadvantage in political and economic spheres. However, this narrative has not been found to foster a stronger sense of common cause and collective action than the WCVN. This may be because the covictimization narrative merely asserts that both genders are victims of gender inequality, without revealing the interconnectedness of their experiences, which may help explain why it did not generate stronger collective-action intentions than the WCVN.

Taken together, existing research suggests that more inclusive victimhood narratives may offer certain advantages, yet no studies have directly examined the effects of the IVN. The IVN may offer a distinct advantage because it encompasses both men’s and women’s victimhood while highlighting the interconnected nature of their role-based constraints. If men come to see that expectations imposed on women simultaneously help produce expectations imposed on men, they may be more likely to view these arrangements as system-level constraints rather than as natural or legitimate role divisions. Importantly, prior studies on related narratives have primarily focused on outcomes such as men’s collective action intentions for women, openness to feminism, identification with feminism, and perceptions of common cause, rather than on whether men come to question the gender-role system itself. The present research extends prior work, which has primarily examined men’s willingness to support women’s causes, by asking whether men can be engaged with gender equality as a shared concern. Specifically, it examines whether the IVN leads men to question gender-role prescriptions for both men and women more strongly than the WCVN. It also extends this literature to a Chinese context, whereas previous research has been conducted primarily with Western samples.

#### 1.1.3. The IVN and the Chinese Context

The broader logic underlying the IVN is not assumed to be unique to China. Across societies, differentiated gender roles can be mutually reinforcing. However, the specific forms and salience of these linked expectations are culturally situated. China provides a relevant context for examining this logic because substantial progress in some domains coexists with persistent inequalities in others. In 2023, women accounted for 49.9% of students in higher education and 50.6% of graduate students, indicating near gender parity in educational access. However, women constituted 43.3% of the employed population and only 26.5% of deputies to the National People’s Congress (National Bureau of Statistics of China, 2025). These patterns suggest that educational gains have not fully translated into equivalent economic and political participation.

Gender inequalities are also embedded in the organization of work and family across both urban and rural China. Employed women spent an average of 649 min on paid and unpaid work per workday, including 495 min on paid work and 154 min on caregiving, cooking, cleaning, and other household tasks; their unpaid work time was approximately twice that of employed men (Office of the Leading Group for the Fourth China Women’s Social Status Survey, 2021). These unequal caregiving responsibilities can restrict women’s occupational opportunities while sustaining the corresponding expectation that men demonstrate economic competence and assume primary provider responsibility. The demographic legacy of the one-child policy has further intensified these pressures. With fewer siblings available to share responsibility for children and aging parents, approximately 60 million working-age Chinese women are estimated to bear medium to high levels of caregiving responsibility (Kwete et al., 2024). Meanwhile, the combination of population-control policies and persistent son preference has contributed to a substantial gender imbalance. The 2020 census recorded 111.3 male births per 100 female births and an overall population sex ratio of 105.07 (National Bureau of Statistics of China, 2021). The resulting male marriage squeeze has increased competition and wealth-accumulation pressure among men and families with sons (Jiang et al., 2014; Wei & Zhang, 2011).

Taken together, these patterns illustrate how gender inequality in contemporary China generates distinct but interconnected constraints within a shared gender system: women face disproportionate caregiving burdens and restricted occupational opportunities, while men are expected to demonstrate economic competence and assume primary provider responsibilities. This makes China a relevant context for testing whether making such interdependence explicit can weaken men’s endorsement of restrictive gender roles.

Accordingly, the present studies primarily operationalized this interdependence through the breadwinner–caregiver division, linking women’s caregiving burdens and career constraints to men’s provider obligations and pressure to achieve occupational success. These culturally salient examples therefore provide a culturally situated test of the IVN in contemporary China.

### 1.2. Men’s Awareness of Gender Discrimination Against Themselves

The present research adopts a structural conceptualization of gender discrimination. Although discrimination can include discrete acts of unequal or hostile treatment, it can also take the form of gender-based role prescriptions that are enforced through social expectations, evaluative standards, and normative pressure. Under this view, discrimination does not require a single identifiable perpetrator or an explicitly hostile act. It can also manifest as the systemic imposition of role-based constraints that limit individuals’ behavioral options on the basis of gender. Accordingly, the present research uses perceived gender discrimination to refer to participants’ recognition of such structural constraints as experienced by women and men, respectively.

The following argument is not intended as a culture-specific claim, but as a broader psychological possibility that the present research examines among Chinese men. Although men may experience such role-based constraints, they generally do not view themselves as victims (Kobrynowicz & Branscombe, 1997). One reason is that discrimination against men is more ambiguous and less readily recognized as gender discrimination, as gender discrimination is more commonly understood as men discriminating against women (Inman & Baron, 1996). In addition, men tend to pay less attention to gender discrimination, whereas women are more likely to perceive it as pervasive and enduring and thus develop a more systematic understanding of it (Inman & Baron, 1996; Jetten et al., 2013). As a result, men may be less likely to interpret their gender-role constraints as forms of gender discrimination.

Moreover, men may also be reluctant to adopt a victim position, because victimhood is often associated with lower status and dependence (Moscovici & Pérez, 2007). Thus, even when men experience role-based constraints, they may interpret these experiences in individualized or defensive ways rather than as products of gender inequality.

At the same time, prior research suggests that non-confrontational narratives acknowledging men’s suffering can promote more constructive responses to gender equality under some conditions (Vázquez et al., 2024). Building on this possibility, the IVN explicitly acknowledges that men are also constrained by gender inequality. Although such acknowledgment does not necessarily guarantee that men will recognize themselves as victims, it may help reframe men’s difficulties as linked to gendered social expectations rather than as purely personal failures. In this sense, the IVN may increase men’s awareness of gender discrimination against themselves. Therefore, we hypothesize the following:

**Hypothesis 1.** 
*Compared to the WCVN, reading the IVN leads men to more readily identify gender discrimination against men.*

### 1.3. Legitimacy of Gender Roles

Unlike the WCVN, which overlooks the impact of gender roles on men, the IVN explicitly addresses the constraints gender roles place on men. Consequently, the IVN may have the potential to reduce men’s perceived legitimacy of gender roles. Prior intervention research suggests that informational interventions can effectively shift beliefs about specific gender-related issues. For example, informing individuals about the negative consequences of gender discrimination after confrontation, beyond simple confrontation, has been shown to increase future regulatory intentions toward gender discrimination (Parker et al., 2018). Educating individuals about structural gender discrimination in STEM fields improves their ability to recognize such discrimination, fostering stronger confrontational attitudes and intentions (Chamberlin & Plant, 2023; Pietri et al., 2017). These interventions increase gender inequality knowledge, raise awareness of gender bias in STEM, and improve self-efficacy in identifying subtle biases. In a similar vein, the IVN may also shift men’s views of gender roles by highlighting their harmful consequences for men.

Beyond highlighting these harmful consequences, the IVN also adopts a social constructionist perspective to explain gender roles, emphasizing that these restrictions are socially imposed. It encourages the understanding that men are not inherently bound to specific behaviors. By examining gender roles from a broader and structural perspective, men are more likely to recognize the universal nature of these constraints, realizing that they affect all men, not just individuals. Such recognition may make existing gender-role arrangements appear less natural or legitimate (Schudson & Gelman, 2023). Similarly, when economic inequality is seen as a structural barrier, individuals are more inclined to recognize its inherent unfairness and support policy reforms (McCall et al., 2017).

In summary, by highlighting the harmful consequences of male gender roles and framing them as socially constructed rather than natural, the IVN may reduce men’s perceived legitimacy of these roles. We hypothesize the following:

**Hypothesis 2a.** 
*Compared to reading the WCVN, men who read the IVN are less likely to legitimize gender roles for men.*

Furthermore, the IVN may reduce men’s perceived legitimacy of gender roles not only for men, but also for women. Compared with the WCVN, the IVN may evoke less defensiveness among men because it does not frame women as the only victims of gender inequality. According to moral typecasting theory, social groups are often cast into the roles of victims and perpetrators (Gray & Wegner, 2009). Under the WCVN, when only women are positioned as victims, men may more easily be cast into the morally suspect role of perpetrator, which can trigger defensiveness and resistance. Prior research suggests that when advantaged group members feel morally rejected or threatened, they may become less open to inequality-reducing change (Hässler et al., 2019, 2022; Sullivan et al., 2012). By acknowledging that men, too, are constrained by gender roles, the IVN may reduce this defensiveness and create a more receptive context for reevaluating gender role arrangements. This may be especially important because acknowledging men’s suffering does not automatically lead men to question existing gender-role arrangements; such recognition may otherwise be interpreted in ways that preserve existing group interests or justify resistance to change.

At the same time, the IVN does not merely acknowledge men’s suffering in isolation. Rather, it emphasizes that men’s and women’s role constraints are interconnected. This may help men understand gender roles as a shared structural problem rather than as separate grievances affecting each gender independently. A shared sense of victimization can improve intergroup relations and stimulate collective action. For example, in the context of the Israeli-Palestinian conflict, inclusive victimhood narratives that describe both sides as victims have been shown to reduce moral defensiveness and promote forgiveness (Adelman et al., 2016; Shnabel et al., 2013). Likewise, increasing awareness of shared experiences of discrimination among marginalized groups can enhance solidarity, foster more positive intergroup attitudes, and increase willingness to support other groups’ causes (Craig & Richeson, 2012; Glasford & Calcagno, 2012; Sanchez, 2008). Applied to the present context, if men come to see that the expectations imposed on women also help sustain the expectations imposed on men, they may become more willing to question the legitimacy of gender roles for both genders, rather than only for themselves. Based on this reasoning, we hypothesize the following:

**Hypothesis 2b.** 
*Compared to reading the WCVN, men reading the IVN are less likely to legitimize gender roles for both men and women.*

## 2. Current Study

The present research examined whether the IVN could engage men with gender equality more effectively than the traditional WCVN. It had two related objectives. First, we tested whether the IVN would increase men’s recognition that they themselves are constrained by gender inequality (H1). Second, we examined whether the IVN would lead men to question restrictive gender roles, first by reducing the perceived legitimacy of roles imposed on men (H2a), and further by extending this rejection to roles prescribed for women as well (H2b).

Study 1 provided an initial test among 200 Chinese men who were randomly assigned to read either the IVN or the WCVN. We assessed whether the IVN increased perceived discrimination against men without reducing perceived discrimination against women, as well as whether it reduced the general legitimacy attributed to the existing gender-role system. Because this general measure did not distinguish between roles prescribed for men and women, Study 2 provided a more differentiated test of H2a and H2b. Participants were assigned to the IVN, WCVN, or a neutral control condition and evaluated specific masculine and feminine prescriptions for both male and female targets. This design allowed us to examine whether the IVN weakened role expectations imposed on men and whether this effect extended to expectations imposed on women.

Across both studies, we reported all experimental conditions, measures, and data exclusion criteria. Sample sizes were determined a priori, and no analyses were conducted before data collection was complete. Data, code, and materials are available at https://osf.io/g63rv/overview (accessed on 22 May 2026).

## 3. Study 1

In Study 1, we compared the effectiveness of the IVN with that of the traditional WCVN in the Chinese context. Specifically, we examined whether exposure to a narrative emphasizing the interconnected victimization of both women and men would reduce participants’ tendency to legitimize the existing gender role system. In the baseline condition, participants were presented with a conventional narrative framing women as the primary victims of gender inequality. Perceived discrimination against women and men, as well as gender role legitimacy, were assessed as dependent variables. We also assessed participants’ appraisals of the narratives, including agreement, perceived falsity, perceived manipulation, and offensiveness, to examine whether the IVN was received more favorably than the WCVN. Comprehensibility and effortfulness were included as reading-experience checks to assess whether any differences between conditions might reflect differences in the accessibility or cognitive demands of the materials.

Although the primary aim of Study 1 was to test whether the IVN was more effective than the WCVN, we also explored whether the effectiveness of the IVN varied as a function of men’s gender-related orientations. Specifically, we examined whether hostile sexism and identification with masculinity moderated the effects of narrative condition. Both constructs have been associated with stronger endorsement of traditional gender roles (Glick & Fiske, 1996; Glick et al., 2015), suggesting that they may shape men’s responses to the narratives that challenge these roles. Some evidence further suggests non-confrontational narratives may be especially effective among men who more strongly adhere to traditional gender roles for some outcomes, although such moderation has not been observed consistently across studies (Vázquez et al., 2024). Accordingly, we treated these moderation analyses as exploratory rather than as a central test of the IVN model.

### 3.1. Method

#### 3.1.1. Participants

The study employed a one-factor between-subjects design, with participants randomly assigned to one of two narrative conditions: the IVN or the WCVN. An a priori power analysis was conducted using G*Power 3.1 (Faul et al., 2007) to determine the minimum required sample size. Based on a similar study comparing the effects of different parental framing (Subašić et al., 2018), we estimated an effect size of *f* = 0.20. With α = 0.05, 1 − β = 0.80, the minimum required sample size for a one-way ANOVA was 200 participants, with 100 participants in each narrative condition. Therefore, we aimed to obtain 200 valid responses.

The study was conducted entirely online through Credamo (https://www.credamo.com/), a Chinese online survey platform. Participants were recruited from Credamo’s online participant pool and completed the questionnaire remotely using their own devices. Eligibility was restricted to Chinese adult men. After eligibility screening, Credamo’s built-in randomization function assigned participants to the IVN or WCVN condition. Recruitment continued until the prespecified total of 200 valid responses was reached after applying the exclusion criteria. Of the 215 respondents recruited, 15 were excluded for failing at least one manipulation or attention check. The final sample comprised 200 participants, including 99 in the IVN condition and 101 in the WCVN condition. Condition-specific participant flow, exclusion rates, and tests of their association with experimental condition for both studies are reported in Table S1 in Supplementary Materials. A sensitivity analysis based on the final analytical sample further indicated that the study had 80% power to detect an effect of at least *f* = 0.20, corresponding to *η*^2^ = 0.04, at the significance level of 0.05.

Participants were compensated 3 CNY (roughly 0.44 USD) for their participation. Their mean age was 31.82 years (*SD* = 9.10). Most held a bachelor’s degree (77.50%), earned between 6000–10,000 CNY monthly (approximately 888–1480 USD; 38.00%), and considered themselves middle-class (i.e., ranked 5th or 6th; 59.00%). Participants were homogeneous across conditions in measured ideological or sociodemographic characteristics (*p*s > 0.10).

#### 3.1.2. Procedure

This study was reviewed and approved by the Ethics Committee of the corresponding author’s university. This approval also covered the subsequent studies reported in this article. All participants provided informed consent before participation.

At the beginning of the questionnaire, participants were asked to report their gender. Those who did not identify as men were informed that they were not the study’s target population and were asked to leave. Participants were then randomly assigned to read one of the two narratives. All participants were informed that the article would present the widely acknowledged opinions of researchers regarding gender bias and discrimination.

The manipulation was achieved through the content of the narratives. Both narratives adopted a social constructionist perspective, framing gender discrimination as a product of social systems rather than individual factors. The IVN presented both men and women as victims of gender inequality, and the WCVN, by contrast, focused solely on women. Both narratives used examples related to the freedom to cry and social roles surrounding caregiving and breadwinning. The IVN described the interconnected limitations faced by both men and women. Emotional expression is constrained differently by gender: men are discouraged from crying, whereas women’s crying is more socially tolerated but may reinforce stereotypes that women are emotionally weak or irrational. In addition, women, as presumed caregivers, were discouraged from pursuing high achievement in school, faced gender-based discrimination at work, and endured the burden of unpaid housework. Meanwhile, men were expected to serve as providers. If they failed to do so, they were viewed as weak and irresponsible, which often led to stress and low self-esteem in unemployed men and stay-at-home fathers. In contrast, the WCVN only mentioned the constraints faced by women. Both narratives concluded by summarizing their main arguments.

After reading the article, participants completed the manipulation checks and also reported two reading-experience checks. Comprehensibility assessed how easy the narrative was to understand, whereas effortfulness assessed how much cognitive effort participants felt was required to read it. These measures were included to examine whether the two narratives differed in accessibility or cognitive demand. The narratives did not differ significantly in comprehensibility (*F*(1, 198) = 1.12, *p* = 0.29, *η*^2^ = 0.01), although the IVN was rated as requiring greater effort (*F*(1, 198) = 9.29, *p* = 0.003, *η*^2^ = 0.04). Participants who failed the manipulation checks were excluded because incorrect responses indicated that they had not understood the narrative content sufficiently for the manipulation to be considered valid. This exclusion rule was determined a priori and applied consistently across conditions.

Participants then completed scales measuring the variables under investigation. The order of items within each scale and the order of some similar variables were randomized. Finally, participants were debriefed and thanked for their participation.

#### 3.1.3. Measures

Unless specified otherwise, all scales ranged from 1 (*Strongly disagree*) to 6 (*Strongly agree*). For multi-item measures, mean scores were computed after reverse coding when necessary, such that higher scores reflected higher levels of the focal construct. Thus, possible composite scores ranged from 1 to 6.

All measures and study materials were administered in Chinese. The Chinese wording of the adapted and author-developed items was reviewed by members of the research team with expertise in social and gender psychology to ensure conceptual clarity and relevance to the intended constructs. The measures were evaluated in the present Chinese sample but have not been independently validated in a separate Chinese sample.

***Manipulation Check.*** Two single-choice questions were used to verify whether participants correctly understood the content of the narrative. First, participants were asked to identify the victim group discussed. Then, they were asked to select the correct description of the relationship between men and women. Participants assigned to the IVN group were expected to select “men and women” and “Both genders are limited by gender bias or discrimination because society requires them to behave oppositely in the same roles”, respectively. Participants assigned to the WCVN group were expected to select “women” and “Women are primarily limited by gender bias or discrimination, while men rarely experience gender discrimination.” Only participants who answered both questions correctly were included.

***Reading-Experience Checks.*** Comprehensibility and effortfulness were each assessed using one author-developed item. Comprehensibility was measured with the item “I could understand the viewpoint expressed in this article,” whereas effortfulness was measured with the item “It took me a relatively long time to read this article.” They were analyzed separately.

***Perceived Discrimination against Women.*** Four items were developed to assess the extent to which participants recognized social discrimination against women. Three items were worded positively (e.g., “Chinese women need to satisfy strict social expectations”), and one item was negatively keyed (“Society does not require women to play certain roles”). The validity and reliability were good (χ^2^ = 0.95, *df* = 2, CFI = 1.00, TLI = 1.01, RMSEA = 0.00; α = 0.82). After reversing the negative-keyed item, mean scores were calculated across items, with higher scores indicating a greater perception of discrimination against women.

***Perceived Discrimination against Men.*** The Perceived Discrimination against Women scale was adapted by changing the subject from “women” to “men” to assess discrimination against men (χ^2^ = 1.13, *df* = 2, CFI = 1.00, TLI = 1.01, RMSEA = 0.00; α = 0.88). After reverse coding the negatively keyed item, items were averaged such that higher scores indicated greater perceived discrimination against men.

***Gender Role Legitimacy.*** Six items were used to measure the extent to which participants legitimize current gender roles. Three items were adapted from the Gender System Justification Scale (Jost & Kay, 2005), and the other three items were author-developed to broaden the initial item pool. A one-factor CFA of all six items indicated poor fit (χ^2^ = 53.05, *df* = 9, CFI = 0.89, TLI = 0.81, RMSEA = 0.16). After removing the item with the lowest factor loading, the resulting five-item model showed substantially improved fit (χ^2^ = 14.48, *df* = 5, CFI = 0.97, TLI = 0.94, RMSEA = 0.10). Example items were as follows: “The current gender roles make everyone happy”; “People should not be required to follow gender roles” (α = 0.81). Mean scores were calculated for the remaining five items, with higher scores indicating greater gender role legitimacy. The complete item wording and additional details regarding item removal are provided in Table S2. Because the five-item version was refined and evaluated within the same sample, evidence supporting its measurement validity remains provisional and requires further validation in an independent sample.

***Narrative Appraisals.*** Four items were developed to measure how the narrative text was appraised. Each appraisal dimension was assessed with a single item because it represented a specific and concrete judgment about the article rather than a broad psychological construct requiring multiple indicators. The four items were therefore scored and analyzed separately rather than combined into a scale. The items were: “I agree with the opinion the article conveyed”, “I do not think the article reflected reality”, “I think this article aimed to lure men into supporting feminism”, and “I felt offended while reading the article”. Higher scores indicated greater agreement, perceived falsity, perceived manipulation, and offensiveness, respectively.

***Hostile Sexism.*** Eleven items from the Hostile Sexism subscale of the Ambivalent Sexism Inventory (Glick & Fiske, 1996) were used to measure participants’ overtly negative attitudes towards women, with three items worded negatively. For example, “Women seek to gain power by getting control over men”. The reliability was good (α = 0.91). Mean scores were calculated after reverse coding the negatively worded items, with higher scores indicating greater hostile sexism.

***Identification with Masculinity.*** Four items were used to measure the extent to which participants identify with masculinity. Two items were adapted from Rivera-Rodriguez et al. (2022), and two items were self-developed. For example, one item was “Being masculine is an important part of who I am”. The validity and reliability were satisfactory (χ^2^ = 4.02, *df* = 2, CFI = 0.99, TLI = 0.98, RMSEA = 0.07; α = 0.79). Mean scores were calculated, with higher scores indicating greater identification with masculinity.

***Sociodemographic Variables.*** The participants reported their age in numeric form, and indicated their level of education (1 = *less than junior high school*, 5 = *master or doctoral degree*), personal monthly income (1 = *between 1000 and 3000 CNY*, 6 = *above 50,000 CNY*), and subjective socio-economic status (1 = *the lowest*, 10 = *the highest*).

***Attention Checks.*** Participants were asked to select a specific option. Those who failed either one of two attention checks were excluded from the analysis.

### 3.2. Results

#### 3.2.1. Descriptive and Correlational Analyses

Descriptive statistics and correlations between all variables were presented in Table 1. In general, the correlation pattern among conceptually related variables was as expected. However, discrimination against men was not associated with other outcome variables. These correlations were obscured by opposite directions across the two narrative conditions (see Table S3 in Supplementary Materials). In the WCVN condition, discrimination against men was associated with lower discrimination against women, higher gender role legitimacy, and more negative appraisal of the narrative. In contrast, in the IVN condition, the correlations were significant in the opposite direction, as the meaning of discrimination against men may be reinterpreted in alignment with feminist perspectives.

#### 3.2.2. Effects of Narrative Condition on Outcome Variables

A one-way between-subjects ANOVA was conducted to compare the effect of narrative (the WCVN vs. the IVN; see Table 2). The results indicated that men reading the IVN acknowledged discrimination against men to a greater extent (*M* = 4.69, *SD* = 0.72) compared to the WCVN (*M* = 2.96, *SD* = 1.18; *F*(1, 198) = 157.60, *p* < 0.001, *η*^2^ = 0.44), and they legitimized the current gender roles less (IVN: *M* = 2.59, *SD* = 0.75; WCVN: *M* = 3.01, *SD* = 0.90; *F*(1, 198) = 12.98, *p* < 0.001, *η*^2^ = 0.06). Also, they appraised the narrative more positively. Specifically, they reported greater agreement (IVN: *M* = 4.91, *SD* = 0.92; WCVN: *M* = 4.08, *SD* = 1.38; *F*(1, 198) = 25.11, *p* < 0.001, *η*^2^ = 0.11), lower perceived falsity (IVN: *M* = 2.07, *SD* = 1.00; WCVN: *M* = 2.85, *SD* = 1.41; *F*(1, 198) = 20.30, *p* < 0.001, *η*^2^ = 0.09), lower perceived manipulation (IVN: *M* = 1.95, *SD* = 0.77; WCVN: *M* = 2.58, *SD* = 1.22; *F*(1, 198) = 19.24, *p* < 0.001, *η*^2^ = 0.09), and lower offensiveness (IVN: *M* = 2.16, *SD* = 0.89; WCVN: *M* = 2.52, *SD* = 1.25; *F*(1, 198) = 5.56, *p* = 0.02, *η*^2^ = 0.03). However, men did not perceive significantly different discrimination against women between conditions (IVN: *M* = 4.36, *SD* = 0.89; WCVN: *M* = 4.17, *SD* = 1.14; *F*(1, 198) = 1.74, *p* = 0.19, *η*^2^ = 0.01).

Assumption checks (Blanca et al., 2018) and robustness analyses (Welch, 1951; Yuen, 1974) are reported in Tables S4–S7. The primary findings were consistent across the conventional, Welch, and Yuen tests, although the difference in offensiveness was not significant under the trimmed-mean analysis.

Because participants in the IVN condition reported greater effortfulness than those in the WCVN condition (*p* = 0.003), we conducted a supplementary ANCOVA controlling for effortfulness to examine whether the main findings could be attributed to differences in perceived reading demand. In a separate set of ANCOVAs, we controlled for participants’ sociodemographic characteristics. The substantive pattern of results remained unchanged in both sets of analyses (see Table S8).

#### 3.2.3. Exploratory Analyses of Ideological Moderation

We further conducted exploratory moderation analyses using PROCESS v4.2 (Hayes, 2022) in SPSS 27 to examine whether the effects of narrative condition varied across ideological orientations. Hostile sexism and identification with masculinity were separately tested as moderators of the effect of narrative on perceived discrimination against men, perceived discrimination against women, and gender role legitimacy.

For perceived discrimination against men, hostile sexism significantly moderated the effect of narrative condition (*b* = −0.59, *SE* = 0.13, *t* = −4.61, *p* < 0.001), whereas identification with masculinity did not (*b* = −0.14, *SE* = 0.17, *t* = −0.82, *p* = 0.42). The IVN significantly increased perceived discrimination against men across most levels of hostile sexism, although this effect tended to become smaller as it increased. To probe this interaction, we used the Johnson–Neyman technique, which identifies the region of a continuous moderator within which the conditional effect of the focal predictor is statistically significant, rather than testing the effect only at selected moderator values (P. O. Johnson & Neyman, 1936; Preacher et al., 2006). Johnson–Neyman analyses indicated that the effect remained significant until hostile sexism reached approximately 1.94 SD above the mean. By contrast, neither ideological variable significantly moderated the effect of narrative condition on perceived discrimination against women (hostile sexism: *b* = 0.22, *SE* = 0.15, *t* = 1.42, *p* = 0.16; identification with masculinity: *b* = 0.01, *SE* = 0.18, *t* = 0.03, *p* = 0.97).

The interaction between hostile sexism and narrative condition did not reach statistical significance for gender-role legitimacy (*b* = −0.24, *SE* = 0.13, *t* = −1.92, *p* = 0.056). The interaction involving identification with masculinity was also nonsignificant (*b* = 0.01, *SE* = 0.14, *t* = 0.08, *p* = 0.94). Because the hostile–sexism interaction did not reach the conventional significance threshold, the subsequent conditional-effect analyses were treated as exploratory. It was found that the IVN significantly reduced gender role legitimacy among men at mean and high levels of hostile sexism (+1 SD), *p*s < 0.001, but not among men low in hostile sexism (−1 SD), *p* = 0.21. Johnson–Neyman analyses further indicated that the effect became significant when hostile sexism was approximately 0.65 SD below the mean or higher. Inspection of the estimated values suggested that this pattern was partly driven by the fact that men at mean and high levels of hostile sexism reported relatively high gender role legitimacy in the WCVN condition, whereas the corresponding values in the IVN condition were comparatively similar across hostile sexism levels. Details of the exploratory analyses can be found in the Supplementary Materials.

### 3.3. Discussion

The results indicated that the IVN could provide additional benefits over the commonly used WCVN, offering direct support for H1 and initial evidence that the IVN reduced the perceived legitimacy of the existing gender-role system. By highlighting the social expectations faced by both men and women, it increased men’s awareness of their connection to gender inequality. Men not only recognized the social expectations imposed on women, but also became more conscious of those placed on themselves. In parallel, the IVN reduced the perceived legitimacy of current gender roles. Furthermore, exploratory analyses also indicated that participants evaluated the IVN more positively on several dimensions. Finally, the IVN performed similarly to the WCVN with respect to men’s recognition of discrimination against women. This may be because the WCVN already effectively communicates the challenges women face, or because such judgments are shaped by social desirability.

The exploratory moderation analyses further clarified the scope of the IVN’s effectiveness. The IVN increased men’s perceived discrimination against men across levels of hostile sexism and identification with masculinity, suggesting that its effect was not limited to a narrow subgroup of already sympathetic participants. At the same time, its effect on gender role legitimacy was more apparent among men higher in hostile sexism. A plausible interpretation is that these men showed relatively higher gender role legitimacy under the WCVN, leaving more room for the IVN to reduce such endorsement. In this sense, the present research extends prior work by showing that acknowledging men’s constraints can weaken the perceived legitimacy of gender roles themselves. Moreover, rather than acknowledging men’s suffering in isolation, the IVN links men’s and women’s constraints within the same gender system. This suggests that framing men’s suffering as intertwined with women’s may help loosen men’s endorsement of traditional gender arrangements while preserving their recognition of women’s disadvantage.

## 4. Study 2

Study 1 found that the IVN reduced men’s overall endorsement of gender role legitimacy but did not specify whether this applied to men’s or women’s roles. Therefore, Study 2 aimed to explore whether the IVN could encourage broader questioning of prescriptive gender roles for both genders. Prescriptive gender stereotypes were adopted as the dependent variable. If the IVN encourages a broader reconsideration of gender-role prescriptions, participants should show lower endorsement of prescriptive stereotypes for both genders compared to the WCVN condition. Conversely, if its effects are limited to men’s own role interests, men would reduce endorsement of male stereotypes but maintain similar views on female stereotypes.

A control condition was added to provide a non-gender-related baseline for interpreting the effects of the two gender narratives. It was not intended to isolate exposure to gender-related content; the primary theoretical comparison remained between the IVN and WCVN. If both the IVN and WCVN conditions reduce stereotype endorsement compared to the control condition, it would suggest that both narratives may weaken endorsement of prescriptive stereotypes. However, if only the IVN condition leads to significantly lower endorsement, while the WCVN condition shows no difference or even higher endorsement, it would indicate that the IVN has a clearer effect on reducing stereotype endorsement, whereas the WCVN may be ineffective or even counterproductive.

### 4.1. Method

#### 4.1.1. Participants

The study employed a 3 (narrative: IVN, WCVN, control) × 2 (target: men, women) × 2 (content: masculinity, femininity) mixed design. Participants were randomly assigned to one narrative condition and were asked to indicate whether men and women should display masculine and feminine traits.

An a priori power analysis was conducted with G*Power 3.1 (Faul et al., 2007) to determine the minimum required sample size. To be conservative, a small effect size (*f* = 0.14) was assumed. Using the repeated-measures within-between interaction option, we set *f* = 0.14, α = 0.05, power (1 − β) = 0.80, three groups, four repeated measurements corresponding to the four Target × Content combinations, a correlation of 0 among repeated measures, and a nonsphericity correction of ε = 1. The analysis yielded a minimum required sample size of 177 participants. We aimed to obtain 270 valid responses from Chinese men through Credamo. Participants were recruited from the online participant pool and completed the questionnaire remotely. After eligibility screening, Credamo’s built-in randomization function assigned participants to the IVN, WCVN, or control condition. Equal quotas of 90 valid responses were set for the three conditions, and recruitment continued until each quota was reached after applying the prespecified exclusion criteria. In total, we recruited 294 respondents and excluded 24 who failed at least one manipulation or attention check. The final analytical sample therefore consisted of 270 participants, with 90 participants in each narrative condition. Using the same specification, a sensitivity analysis indicated that the study had 80% power to detect a three-way interaction effect of at least *f* = 0.11, corresponding to *η*^2^ = 0.01, at the significance level of 0.05. Participants were compensated 3 CNY for their participation.

The mean age of participants was 29.87 years (*SD* = 7.88). The majority held a bachelor’s degree (72.60%), earned between 6000–10,000 CNY monthly (approximately 888–1480 USD; 39.60%) and identified as middle-class (56.60%). Fourteen participants identified as sexual minorities (5.20%), and three did not report (1.10%). Sexual orientation was not assessed in Study 1. Participants did not differ significantly in sociodemographic characteristics across conditions (*p*s > 0.33).

#### 4.1.2. Procedure

The procedure of Study 2 was similar to that of Study 1. At the beginning of the questionnaire, participants reported their gender. They were then randomly assigned to one of the three narrative conditions. The manipulation materials differed from those used in Study 1 in three respects. First, we revised the IVN to make the interdependence of gendered expectations more explicit. For example, after describing the constraints placed on women as caregivers, we added a statement linking the role restrictions faced by both genders: “In fact, this is not only discrimination against women but also discrimination against men.” This highlighted that the expectation for women to be caregivers inherently reinforced expectations for men to be providers, illustrating how social expectations on one gender simultaneously imposed restrictions on the other. Second, we shortened the narratives to approximately 400 Chinese characters to better maintain participants’ attention. The content of the WCVN was carefully aligned with the revised IVN. Third, we introduced a control condition to provide a non-gender-related baseline. The control article discussed scholars’ consensus on the importance of sleep. To ensure structural consistency, it followed the same format as the experimental narratives (i.e., introduction, two arguments with a corresponding example, and conclusion).

To assess participants’ reading experience, they also reported how understandable they found the text and how much effort it required to read, indexed by one item on comprehensibility and one item on effortfulness. The three conditions did not differ significantly in either comprehensibility (*F*(2, 267) = 1.01, *p* = 0.33, *η*^2^ = 0.01), or effortfulness (*F*(2, 267) = 0.56, *p* = 0.55, *η*^2^ = 0.004), suggesting that the materials were comparable in reading experience.

After reading the materials, participants completed the manipulation checks. They then responded to scales measuring prescriptive gender stereotypes for men and women, with the order of target gender randomized. Masculinity and femininity trait words were also randomly mixed within the scales. Finally, participants were debriefed and thanked for their participation.

#### 4.1.3. Measures

All materials and measures were administered in Chinese.

***Manipulation Checks.*** Participants in the IVN and WCVN conditions completed the same manipulation checks as in Study 1. In the control condition, they identified the unmentioned benefit of sleep in the text. Those who failed any check were excluded.

***Prescriptive Gender Stereotypes.*** Eighteen personality traits were selected from the Chinese adaptation (Zhao & Zhang, 2018) of the Bem Sex Role Inventory (Bem, 1974) to assess participants’ endorsement of prescriptive gender stereotypes. To reduce questionnaire length while retaining balanced coverage of the original trait domains, 9 traits were selected from the original 15 masculine traits and 15 feminine traits respectively. Example masculine traits included ambitious, rational, and extroverted, and example feminine traits included compassionate, kind, and tender. Participants rated the extent to which men and women should possess each trait. Their responses were used to construct four nine-item subscales assessing prescriptions concerning women’s femininity, women’s masculinity, men’s femininity, and men’s masculinity. Mean scores were calculated across the nine items in each subscale. Responses ranged from 1 (*Strongly disagree*) to 6 (*Strongly agree*), with higher scores indicating stronger endorsement of the corresponding prescriptive stereotype. The four subscales showed acceptable to good internal structure and reliability (Stereotype_WomenFem_: χ^2^ = 64.84, *df* = 26, CFI = 0.96, TLI = 0.94, RMSEA = 0.07, α = 0.86; Stereotype_WomenMasc_: χ^2^ = 45.87, *df* = 24, CFI = 0.98, TLI = 0.97, RMSEA = 0.06, α = 0.87; Stereotype_MenFem_: χ^2^ = 49.36, *df* = 26, CFI = 0.98, TLI = 0.97, RMSEA = 0.06, α = 0.87; Stereotype_MenMasc_: χ^2^ = 50.55, *df* = 24, CFI = 0.97, TLI = 0.95, RMSEA = 0.05, α = 0.85).

***Sociodemographic Characteristics.*** Participants reported their age (in years), education level (1 = less than junior high school, 5 = master or doctoral degree), monthly income (1 = between 1000 and 3000 CNY, 6 = above 50,000 CNY), subjective socio-economic status (1 = the lowest, 10 = the highest), and sexual orientation (0 = heterosexual, 1 = non-heterosexual, 2 = unknown).

***Attention Checks.*** Participants completed two attention checks requiring a specific response. Those who failed either were excluded.

### 4.2. Results

#### 4.2.1. Descriptive and Correlational Analyses

Descriptive statistics and correlations between all variables were presented in Table 3.

#### 4.2.2. Effects of Narrative Condition on Outcome Variables

A 3 (Narrative) × 2 (Target) × 2 (Content) mixed-design ANOVA was conducted, and pairwise comparisons reported in this section were Bonferroni-adjusted. Significant main effects were obtained for Narrative (*F*(2, 267) = 5.17, *p* = 0.006, *η*^2^*_p_* = 0.04; *M_IVN_* = 4.33, *SE* = 0.05; *M_WCVN_* = 4.54, *SE* = 0.06; *M_control_* = 4.54, *SE* = 0.05), Target (*F*(1, 267) = 43.84, *p* < 0.001, *η*^2^*_p_* = 0.14; *M_men_* = 4.55, *SE* = 0.03; *M_women_* = 4.39, *SE* = 0.04), and Content (*F*(1, 267) = 4.45, *p* = 0.04, *η*^2^*_p_* = 0.02; *M_masculinity_* = 4.45, *SE* = 0.03; *M_femininity_* = 4.49, *SE* = 0.03). Pairwise comparisons indicated that men who read the IVN were less likely to endorse prescriptive gender stereotypes in general compared to the WCVN (*p* = 0.02) and control conditions (*p* = 0.02), whereas the difference between the WCVN condition and the control condition was negligible (*p* = 1.00).

Importantly, neither the Narrative × Target interaction (*F*(2, 267) = 0.95, *p* = 0.39, *η*^2^*_p_* = 0.01) nor the Narrative × Content interaction (*F*(2, 267) = 1.98, *p* = 0.14, *η*^2^*_p_* = 0.01) was significant. The Narrative × Target × Content interaction also did not reach statistical significance (*F*(2, 267) = 2.71, *p* = 0.07, *η*^2^*_p_* = 0.02). Thus, there was no clear evidence that the effect of narrative condition varied according to target gender or stereotype content, either separately or jointly.

Independent of Narrative, Target × Content was significant (*F*(1, 267) = 382.61, *p* < 0.001, *η*^2^*_p_* = 0.59). Participants endorsed the view that men should display masculine traits more strongly (*M* = 4.94, *SE* = 0.04) than feminine traits (*M* = 4.15, *SE* = 0.04), *p* < 0.001. Similarly, participants showed greater agreement that women should display feminine traits (*M* = 4.84, *SE* = 0.04) compared to masculine traits (*M* = 3.95, *SE* = 0.05), *p* < 0.001.

To provide further information about the pattern across specific role prescriptions, we conducted exploratory follow-up analyses of the simple effect of Narrative within each Target × Content cell. Because the three-way interaction was not significant, these analyses should not be interpreted as evidence that the Narrative effect differed reliably across the four cells.

**Figure 1:**
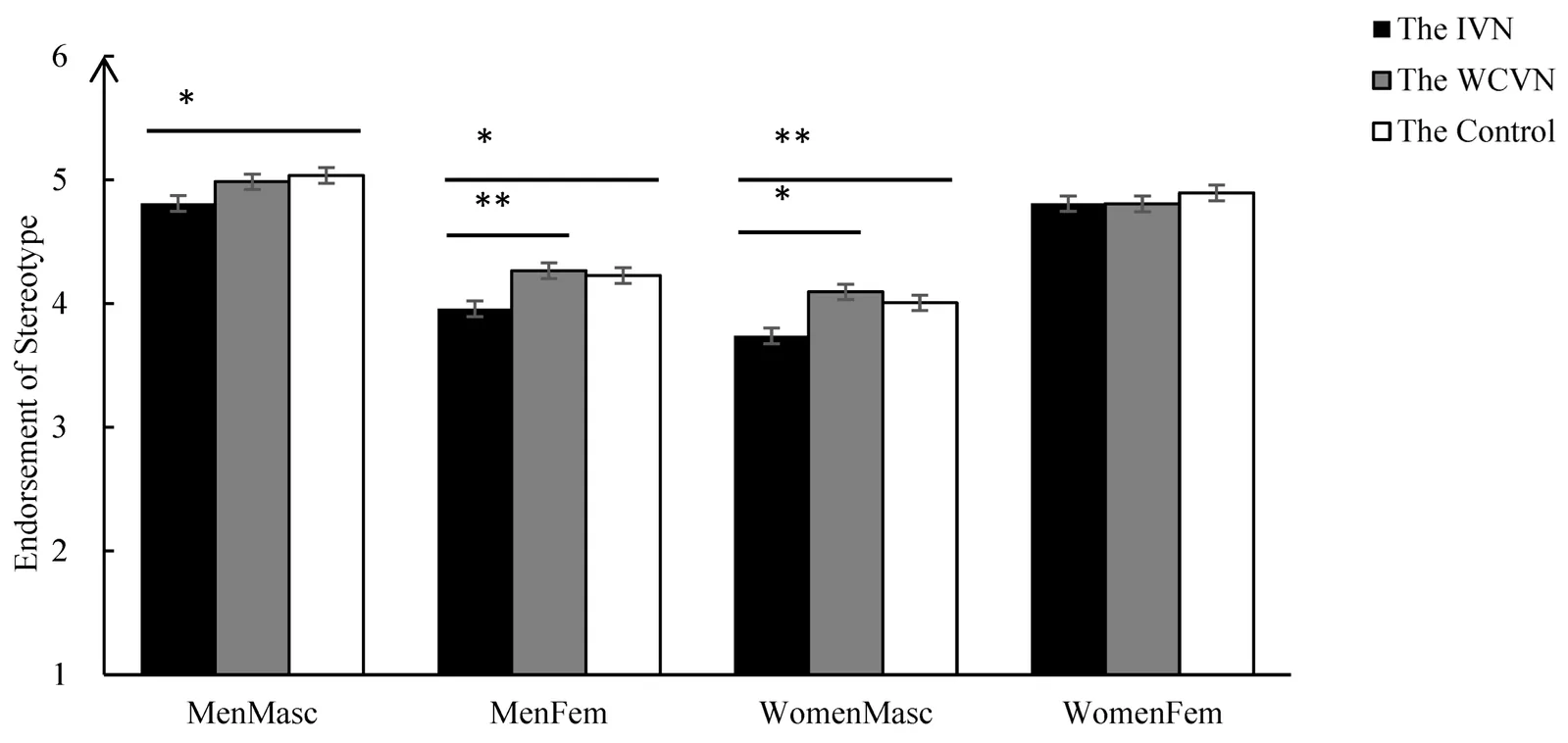
Mean endorsement of stereotype as a function of target, content, and narrative. *Note*. Error bars represent standard errors. * *p* < 0.05; ** *p* < 0.01.

The exploratory effect of Narrative was significant for prescriptions concerning women should display masculine traits (*F*(2, 267) = 5.75, *p* = 0.004, *η*^2^*_p_* = 0.04); prescriptions that men should display masculine traits, *F*(2, 267) = 3.51, *p* = 0.03, *η*^2^*_p_* = 0.03; and prescriptions that men should display feminine traits, *F*(2, 267) = 5.33, *p* = 0.01, *η*^2^*_p_* = 0.04. For prescriptions concerning women’s masculinity and men’s femininity, endorsement was lower in the IVN condition than in both the WCVN and control conditions, adjusted *ps* < 0.05. For prescriptions concerning men’s masculinity, the IVN condition was lower than the control condition, *p* = 0.04, but did not differ significantly from the WCVN condition, *p* = 0.15. The WCVN and control conditions did not differ significantly in these cells. Narrative did not significantly affect prescriptions that women should display feminine traits, *F*(2, 267) = 0.50, *p* = 0.61, *η*^2^*_p_* = 0.004. Means, standard deviations, standard errors, and 95% confidence intervals for all Target × Content cells are reported in Table S9 in the Supplementary Materials. Figure 1 presents the cell-level results.

Assumption checks and sensitivity analyses (Games & Howell, 1976; Hancock & Klockars, 1996) are reported in Tables S10–S15. And the covariate-adjusted analyses produced the same overall pattern (see Figure S1).

### 4.3. Discussion

The findings suggest that the IVN may encourage a broader reconsideration of prescriptive gender roles. Men exposed to the IVN endorsed these stereotypes less strongly overall than those in the WCVN and control conditions. None of the interactions involving Narrative reached statistical significance, providing no clear evidence that this effect depended on target gender or stereotype content. Exploratory analyses showed lower endorsement of prescriptions concerning men’s masculinity and femininity and women’s masculinity, but not women’s femininity. However, because the three-way interaction was not significant, this pattern does not establish that the IVN was reliably less effective for women’s femininity and should be interpreted cautiously. Taken together, these results support H2a and provide qualified support for H2b.

One possible explanation for the exploratory cell-specific pattern is that the association between women and feminine traits may be especially deeply ingrained and therefore less responsive to a brief narrative intervention. This interpretation is consistent with prior research indicating that gender stereotypes have not changed uniformly over time and that stereotypes concerning women’s femininity may have become more pronounced (Eagly et al., 2020; Haines et al., 2016). Future research should examine whether this pattern can be replicated and whether the effects of the IVN vary across specific gender-role expectations.

## 5. General Discussion

Gender-inequality narratives often center women’s victimization, whereas male-victimhood narratives may position men’s grievances against women and gender equality. The IVN addresses this tension by framing men’s and women’s constraints as interconnected consequences of the same gender-role system. In Study 1, the IVN increased perceived discrimination against men without reducing perceived discrimination against women, supporting H1, and also reduced the perceived legitimacy of the existing gender-role system. Study 2 extended this test from general evaluations of gender roles to specific prescriptions about how men and women should behave. Men exposed to the IVN endorsed prescriptive gender stereotypes less strongly overall than those in the WCVN and control conditions, and none of the interactions involving Narrative reached statistical significance. Exploratory analyses suggested lower endorsement of prescriptions concerning men’s masculinity and femininity and women’s masculinity, but not women’s femininity. Because this cell-specific pattern was exploratory and the three-way interaction was not significant, it should be interpreted cautiously. Taken together, the findings support H2a and provide qualified support for H2b. The WCVN and control conditions did not differ significantly, suggesting that a women-centered victimhood framing alone may be insufficient to prompt men to reconsider restrictive gender-role expectations. Thus, framing gender inequality as an interconnected system of constraints may be more effective than a women-centered victimhood framing in increasing men’s recognition of constraints affecting themselves and weakening their endorsement of restrictive gender arrangements.

The present research broadens the literature on men’s engagement with gender equality by shifting the focus from whether men are willing to support women’s causes to whether they can recognize gender equality as a shared concern. Prior allyship research has generally positioned men as members of an advantaged group who may choose to act on behalf of women (Drury & Kaiser, 2014; Stewart, 2017; Thomas, 2024). The IVN instead presents men as also constrained by the gender-role system and asks whether recognizing these constraints can lead them to question a system that disadvantages women while also restricting themselves. Such recognition, however, does not necessarily produce support for gender equality. From a social identity perspective (Tajfel & Turner, 1979), men might reject the victim position because of its association with lower status, engage in competitive victimhood by recognizing men’s disadvantages while denying women’s, or acknowledge that both genders suffer while still resisting changes to the existing system (Lisnek et al., 2022; Moscovici & Pérez, 2007; Sullivan et al., 2012). However, the observed attitudinal pattern was not consistent with these possibilities. The IVN increased recognition of discrimination against men without reducing recognition of discrimination against women and was accompanied by lower gender-role legitimacy and stereotype endorsement. Thus, at the attitudinal level examined here, recognizing constraints affecting men did not necessarily take a competitive form or reinforce existing gender arrangements. The exploratory moderation findings further indicate that the IVN’s effects were evident among men with relatively high levels of hostile sexism, whereas identification with masculinity did not consistently moderate these effects. Although preliminary, this pattern suggests that neither strong identification with masculinity nor hostile sexist attitudes necessarily preclude men from reconsidering existing gender arrangements.

A second contribution concerns how male victimhood is framed. Discussing men’s victimhood can be risky, as it is often linked to negative outcomes for male audiences, such as less collective guilt (Sullivan et al., 2012) and reduced willingness to combat sexual assault (Lisnek et al., 2022). A related pattern emerged in Study 1: among men who read the WCVN, greater perceived discrimination against men was associated with attitudes less consistent with gender equality. By contrast, the IVN situated men’s constraints within the same gender-role system that constrains women, and the resulting attitudinal pattern was not competitive: increased recognition of discrimination against men coexisted with recognition of discrimination against women and lower endorsement of restrictive gender arrangements. These findings suggest that the consequences of discussing male victimhood depend partly on whether men’s and women’s concerns are framed as competing or interconnected.

Interconnected framing may also help men recognize a shared stake in gender equality, because changing constraints imposed on women may simultaneously weaken complementary expectations imposed on men. However, merely emphasizing shared victimization may be insufficient. Hardacre and Subašić (2022) found that a shared-victimhood narrative did not produce more gender-equal behavior than a women-centered narrative. One possibility is that recognizing common suffering does not necessarily generate shared goals or direct attention toward the gender-role system that sustains both groups’ constraints. Future research should explore the mechanisms of the IVN more thoroughly to identify key psychological processes, and propose alternative interventions that could activate these processes.

Finally, the downstream outcomes of the IVN require further investigation. It is possible that the IVN can reduce men’s endorsement of zero-sum beliefs regarding gender. Prior research has demonstrated that zero-sum beliefs—for example, those between consumers and companies—can be diminished through deeper cognitive reflection (Bhattacharjee et al., 2017; S. G. B. Johnson et al., 2022). Similarly, the IVN may encourage men to support systemic change that liberates both genders from restrictive roles, rather than simply engaging in collective action to defend the status quo (Becker, 2020). Future research could examine whether this framing reduces zero-sum beliefs about gender and increases support for systemic change.

The broader applicability of these findings should be interpreted as a culturally situated test of a potentially general relational logic rather than as evidence of cross-cultural equivalence. The underlying logic may nevertheless extend beyond China. Western research similarly documents prescriptive expectations of male agency and female communality, together with an asymmetric transformation in which women’s movement into paid employment has advanced more rapidly than men’s movement into caregiving and domestic roles (Croft et al., 2015, 2021). However, the concerns through which gendered constraints become salient differ across contexts. In China, men’s provider expectations are closely tied to marriage, housing, and family responsibility, with demographic imbalance further intensifying economic pressure on men and their families (Wei & Zhang, 2011). In Western male-victimhood discourse, recurring concerns include child custody, boys’ educational difficulties, male suicide, and insufficient social support for men (Schmitz & Kazyak, 2016). Despite these differences, adversarial male-victimhood narratives in both contexts commonly position men’s concerns against women or feminism and frame gender relations in zero-sum terms (Dickel & Evolvi, 2023; Yang et al., 2023). The present Chinese studies suggest that the IVN may offer an alternative by linking locally relevant male concerns to corresponding constraints on women within the same gender system. Research in other cultural contexts could therefore test whether the IVN can reframe locally relevant male concerns in similar ways. Thus, although the IVN’s relational logic may extend across cultures, the concerns through which it is communicated and tested should be culturally situated.

## 6. Limitations and Future Directions

Several limitations should be acknowledged. First, both studies assessed self-reported attitudes immediately after a single online exposure. The findings therefore demonstrate short-term attitudinal effects, but not whether these effects persist or translate into allyship, intergender cooperation, or behavioral commitment. Longitudinal and behavioral research is needed to examine the durability and downstream consequences of the IVN.

Second, the narratives were deliberately matched in structure and tone to isolate the effect of framing men’s and women’s constraints as interconnected. This experimental control may limit ecological validity, as naturally occurring gender-inequality narratives vary in tone and content. Moreover, some feminists have already begun to incorporate elements of the IVN (Mikołajczak & Becker, 2022), making it possible to compare the real-world effects of IVN and WCVN. Future studies should examine naturally occurring narratives through text analysis or field experiments.

Third, both studies included only Chinese men and did not assess marital status, parental status, or urban–rural residence, all of which may shape exposure to gendered obligations and responses to the IVN. The findings also do not establish cross-cultural generalizability; culturally adapted versions should be tested across societies and demographic groups.

Fourth, participants who failed the prespecified manipulation or attention checks were excluded after random assignment. Such exclusions may introduce bias. An intention-to-treat analysis was not possible because Credamo does not retain item-level responses once a submission is manually rejected. The findings should therefore be interpreted as per-protocol effects among participants who passed the checks.

Finally, the cell-specific findings in Study 2 were exploratory because the three-way interaction did not reach conventional significance. Their replicability and variation across specific gender-role prescriptions require further investigation.

## 7. Conclusions

The present research introduced the IVN as a way of communicating gender inequality that links men’s and women’s constraints without equating their severity or positioning their concerns in competition. Across two online experiments with Chinese men, the IVN increased recognition of discrimination against men without reducing recognition of discrimination against women and weakened endorsement of restrictive gender arrangements. Study 2 showed an overall reduction in prescriptive stereotypes, with no significant interactions involving narrative condition; accordingly, the findings support H2a and provide qualified support for H2b.

For gender studies, these findings suggest that acknowledging men’s constraints need not displace attention from women’s structural disadvantage or reinforce adversarial male-victimhood claims. Rather, the consequences of discussing male victimhood may depend on whether it is framed competitively or as part of an interconnected gender-role system. For Chinese social psychology, the findings show how locally salient pressures on men, particularly provider and family obligations, can be understood in relation to the caregiving constraints imposed on women, thereby framing gender equality as a shared concern. Future research should test whether these immediate attitudinal effects persist and translate into cooperation, allyship, and behavioral support for gender equality.

## Figures and Tables

**Table 1:** Descriptive Statistics and Correlations among Variables for Study 1.

**Variable**		***M* (*SD*)**		**1**	**2**	**3**	**4**	**5**	**6**	**7**
1	Narrative ^a^	–		1						
2	Discrimination against Men	3.82 (1.30)		0.67 **	1					
3	Discrimination against Women	4.26 (1.02)		0.09	0.09	1				
4	Gender Role Legitimacy	2.80 (0.85)		−0.25 **	−0.11	−0.44 **	1			
5	Agreement	4.49 (1.24)		0.34 **	0.07	0.36 **	−0.35 **	1		
6	Falsity	2.47 (1.28)		−0.31 **	−0.13	−0.36 **	0.37 **	−0.77 **	1	
7	Feminist Manipulation	2.27 (1.07)		−0.30 **	0.09	−0.34 **	0.34 **	−0.48 **	0.45 **	1
8	Offensiveness	2.35 (1.10)		−0.17 *	0.08	−0.17 *	0.15 *	−0.67 **	0.62 **	0.46 **
9	Comprehensibility	5.09 (0.82)		0.07	0.02	0.30 **	−0.27 **	0.40 **	−0.42 **	−0.25 **
10	Effortfulness	3.59 (1.21)		0.21 **	0.18 *	0.12	−0.01	0.04	−0.05	0.00
11	Hostile Sexism	3.15 (0.94)		0.05	0.36 **	−0.21 **	0.16 *	−0.16 *	0.20 **	0.45 **
12	Identification with Masculinity	4.59 (0.84)		−0.06	−0.02	−0.17 *	0.25 **	0.04	0.04	0.22 **
13	Age	31.82 (9.10)		−0.04	−0.15 *	−0.05	0.04	−0.13	0.16 *	−0.03
14	Education	3.93 (0.55)		0.04	0.05	0.04	−0.02	−0.05	0.01	−0.02
15	Income	3.03 (1.07)		−0.10	−0.14 *	−0.08	0.15 *	−0.13	0.11	−0.05
16	sSES ^b^	5.28 (1.37)		−0.06	−0.12	−0.05	0.05	0.01	−0.02	−0.17 *
**Variable**		**8**	**9**	**10**	**11**	**12**	**13**	**14**	**15**	**16**
8	Offensiveness	1								
9	Comprehensibility	−0.31 **	1							
10	Effortfulness	0.03	−0.20 **	1						
11	Hostile Sexism	0.19 **	−0.13	0.05	1					
12	Identification with Masculinity	−0.07	−0.05	−0.09	0.26 **	1				
13	Age	−0.01	−0.22 **	−0.09	−0.22 **	−0.03	1			
14	Education	0.00	0.04	0.01	−0.10	−0.16 *	−0.10	1		
15	Income	−0.02	−0.07	−0.05	−0.28 **	−0.12	0.44 **	0.16 *	1	
16	sSES ^b^	−0.10	0.00	−0.10	−0.19 **	0.01	0.25 **	0.20 **	0.43 **	1

*Note.* ^a^ Narrative was a binary variable (0 = WCVN, 1 = IVN). ^b^ sSES represents subjective socioeconomic status. * *p* < 0.05; ** *p* < 0.01.

**Table 2:** One-way ANOVA Analysis of Narrative Effect on Outcome Variables.

Outcome Variable	Narrative		*F*	*p*	*η* ^2^
	IVN	WCVN			
Discrimination against Men	4.69 (0.72)	2.96 (1.18)	157.60	<0.001	0.44
Discrimination against Women	4.36 (0.89)	4.17 (1.14)	1.74	0.19	0.01
Gender Role Legitimacy	2.59 (0.75)	3.01 (0.90)	12.98	<0.001	0.06
Agreement	4.91 (0.92)	4.08 (1.38)	25.11	<0.001	0.11
Falsity	2.07 (1.00)	2.85 (1.41)	20.30	<0.001	0.09
Manipulation	1.95 (0.77)	2.58 (1.22)	19.24	<0.001	0.09
Offensiveness	2.16 (0.89)	2.52 (1.25)	5.56	0.02	0.03

**Table 3:** Descriptive Statistics and Correlations among Variables for Study 2.

Variable		*M* (*SD*)	1	2	3	4	5	6	7	8	9	10	11	12
1	Narrative ^a^	–	1											
2	Stereotype_MenMasc_ ^b^	4.94 (0.61)	−0.15 *	1										
3	Stereotype_MenFem_ ^c^	4.15 (0.70)	−0.16 **	0.32 **	1									
4	Stereotype_WomenMasc_ ^d^	3.95 (0.74)	−0.15 *	0.41 **	0.69 **	1								
5	Stereotype_WomenFem_ ^e^	4.84 (0.67)	−0.05	0.68 **	0.20 **	0.29 **	1							
6	Comprehensibility	5.26 (0.67)	−0.01	0.15 *	0.00	−0.04	0.18 **	1						
7	Effortfulness	3.78 (1.04)	0.07	0.08	0.11	0.06	0.10	−0.11	1					
8	Age	29.87 (7.88)	0.05	0.04	−0.04	−0.02	0.10	−0.12	−0.05	1				
9	Education	3.91 (0.67)	0.03	0.01	−0.11	−0.04	0.06	0.15 *	0.07	−0.12 *	1			
10	Income	2.90 (1.10)	0.02	0.13 *	−0.09	−0.03	0.11	0.10	−0.02	0.43 **	0.26	1		
11	sSES ^f^	5.22 (1.48)	−0.05	0.14	−0.05	−0.03	0.14 *	0.12 *	−0.05	0.13 *	0.23	0.43	1	
12	Sexual Orientation ^g^	–	0.09	0.02	0.06	0.08	0.03	0.01	0.02	0.01	−0.15	−0.06	−0.16	1

*Note.* ^a^ Narrative was a three-level variable (0 = Control, 1 = WCVN, 2 = IVN). ^b^ StereotypeMenMasc refers to the endorsement of prescriptive gender stereotypes regarding men’s masculinity. ^c^ StereotypeMenFem refers to the endorsement of prescriptive gender stereotypes regarding men’s femininity. ^d^ StereotypeWomenMasc refers to the endorsement of prescriptive gender stereotypes regarding women’s masculinity. ^e^ StereotypeWomenFem refers to the endorsement of prescriptive gender stereotypes regarding women’s femininity. ^f^ sSES represents subjective socioeconomic status. ^g^ Sexual orientation was coded as 0 = heterosexual, 1 = non-heterosexual, and 2 = unknown. * *p* < 0.05; ** *p* < 0.01.

## Data Availability

The data, results, and experimental materials of Studies 1 and 2 are available at the following link: https://osf.io/g63rv/overview (accessed on 22 May 2026).

## Supplementary Materials

The following supporting information can be downloaded at: https://www.mdpi.com/article/10.3390/bs16091609/s1, Table S1: Participant Flow and Exclusions by Experimental Condition; Table S2: Items of the Gender Role Legitimacy Measure in Study 1; Table S3: Correlations among Dependent Variables across Two Conditions in Study 1; Table S4: Tests of Homogeneity of Variance for Outcome Variables in Study 1; Table S5: Tests of Normality for Outcome Variables in Study 1; Table S6: Conventional, Welch, and Yuen’s 20% Trimmed-Mean Tests of the Narrative Effect in Study 1; Table S7: Distribution of Offensiveness Ratings by Narrative Condition in Study 1; Table S8: One-way ANCOVA Analyses of Narrative Effect on Outcome Variables in Study 1; Table S9: Means, Standard Deviations, and 95% Confidence Intervals for All 12 Narrative × Target × Content Cells in Study 2; Table S10: Assumptions of the Mixed-Design Analysis of Variance in Study 2; Table S11: Tests of Homogeneity of the Model Error Strata for Study 2; Table S12: Tests of Normality of the Model Error Strata for Study 2; Table S13: Conventional and Welch Omnibus Tests of Narrative Effects Overall and Within Each Cell in Study 2; Table S14: Games–Howell Pairwise Comparisons of Narrative Conditions Overall and Within Each Cell in Study 2; Table S15: Randomization-Based Sensitivity Analyses of the Study 2 Mixed Design; Figure S1: Mean Stereotype Endorsement as a Function of Target, Content, and Narrative.
