# Supplementary material for: The Intertwined Victimhood Narrative and the Challenge to Chinese Men’s Gender Role Legitimacy
Source: Behav Sci (Basel). 2026 Sep 9;16(9):1609. doi: 10.3390/bs16091609 (PMC13603985; doi:10.3390/bs16091609)
Supplement: Supplementary file 1 [file behavsci-16-01609-s001.zip › behavsci-4365048-supplementary.pdf]

Supplementary Materials

The Intertwined Victimhood Narrative and the Challenge to Chinese Men's Gender Role Legitimacy

Table of Contents

Participant Flow and Exclusions

Supplementary Analyses in Study 1

- Development and Refinement of the Gender Role Legitimacy Measure
- Conditional Correlation Analyses
- Assumption Checks and Robustness Analyses
- One-way Between-subject ANCOVA
- Exploratory Moderation Analyses of Ideological Variables

Supplementary Analyses in Study 2

- Descriptive Statistics for the 3 × 2 × 2 Design
- Assumption Checks and Robustness Analyses
- Three-way Mixed-design ANCOVA

Participant Flow and Exclusions

Recruitment in each study continued until the prespecified target number of valid responses was reached after applying the a priori exclusion criteria. Participants who failed at least one prespecified eligibility, manipulation, or attention check were excluded and replaced during recruitment. Table S1 summarizes the numbers entering the study and assigned to a condition, excluded, and included in the final analyses in each condition.

Chi-square tests of independence were conducted to examine whether exclusion status was associated with experimental condition. In Study 1, exclusion status was not significantly associated with narrative condition, $\chi^2(1, 215) = 1.65$, $p = .20$, Cramér's V = .09. Specifically, 10 of 109 respondents in the IVN condition (9.2%), and 5 of 106 respondents in the WCVN condition (4.7%), were excluded. In Study 2, exclusion status was also not significantly associated with experimental condition, $\chi^2(2, 294) = 0.92$, $p = .63$, Cramér's V = .06. Specifically, 10 of 100 respondents in the IVN condition (10.0%), 6 of 96 respondents in the WCVN condition (6.3%), and 8 of 98 respondents in the control condition (8.2%), were excluded. Thus, there was no evidence that exclusion rates differed systematically across experimental conditions in either study.

Table S1. *Participant Flow and Exclusions by Experimental Condition*

Flow stage	Study 1			Study 2			
	IVN	WCVN	Total	IVN	WCVN	Control	Total
Entered and assigned to a condition	109	106	215	100	96	98	294
Excluded, n (%)	10 (9.2%)	5 (4.7%)	15 (7.0%)	10 (10.0%)	6 (6.3%)	8 (8.2%)	24 (8.2%)
Included in final analyses	99	101	200	90	90	90	270

Note. Percentages are calculated within condition; percentages in the Total column are calculated across all respondents. IVN = Intertwined Victimhood Narrative; WCVN = Women-Centric Victimhood Narrative.

Supplementary Analyses in Study 1

Development and Refinement of the Gender Role Legitimacy Measure

The initial Gender Role Legitimacy measure consisted of six items. Three items were adapted from the Gender System Justification Scale (Jost & Kay, 2005), and three additional items were developed by the authors to broaden the initial item pool. All items were administered in Chinese. The Chinese wording of the adapted and newly developed items was reviewed by members of the research team with expertise in social and gender psychology to ensure conceptual clarity and relevance to the focal construct.

A one-factor confirmatory factor analysis of the six-item measure showed poor fit ($\chi^2 = 53.05$, df = 9, CFI = .89, TLI = .81, RMSEA = .16). Item 5, “Only by liberating people from the current gender roles can everyone live their true selves,” had the lowest factor loading of 0.38, and was removed. In addition to its relatively low loading, this item focused more on personal authenticity following liberation from gender roles than on whether the existing gender-role system was legitimate or should be maintained, making it somewhat less consistent with the content of the other items. See Table S2 for details.

After removing Item 5, the one-factor model showed substantially improved, although mixed, fit ($\chi^2 = 14.48$, df = 5, CFI = 0.97, TLI = 0.94, RMSEA = 0.10). Items 4 and 6 were reverse-scored, and mean scores were calculated across the five retained items. Higher scores indicated greater perceived legitimacy of current gender roles. The five-item measure showed acceptable internal consistency in the present sample ($\alpha = .81$). The measure was evaluated in the present Chinese sample but has not been independently validated in a separate Chinese sample.

Table S2. *Items of the Gender Role Legitimacy Measure in Study 1*

No.	Item wording	Source	Status
1	The current gender roles make everyone happy.	Adapted	Retained
2	The current division of gender roles generally operates as it should.	Adapted	Retained
3	The current division of gender roles is the most reasonable.	Adapted	Retained
4	Gender roles need to be radically restructured.	Developed	Retained
5	Only by liberating people from the current gender roles can everyone live their true selves.	Developed	Removed
6	People should not be required to follow gender roles.	Developed	Retained

Note. Items were administered in Chinese. Item 5 was removed because it had the lowest factor loading and was less closely aligned with the perceived legitimacy and maintenance of the existing gender-role system.

Conditional Correlation Analyses

When comparing the correlations across the two conditions, notable differences emerged, particularly in the associations involving discrimination against men (see Table S3). In the Women-Centric Victimhood Narrative (WCVN) condition, discrimination against men was associated with lower discrimination against women, higher levels of perceived legitimacy of gender roles, and a more negative appraisal of the narrative. Conversely, in the Intertwined Victimhood Narrative (IVN) condition, discrimination against men was not significantly associated with manipulation, or offensiveness. Instead, it was linked to higher discrimination against women, lower perceived legitimacy of gender roles, and a more positive appraisal of the narrative. These findings suggest that the correlations between discrimination against men and other variables were in opposite directions in the two conditions, highlighting the distinct impacts of the two narrative conditions on participants' perceptions.

Table S3. *Correlations among Dependent Variables across Two Conditions in Study 1*

Variables		1	2	3	4	5	6	7
1	Discrimination against Men	1	-.24*	.25*	-.41**	.27**	.62**	.43**
2	Discrimination against Women	.62**	1	-.48**	.44**	-.46**	-.42**	-.22*
3	Gender Role Legitimacy	-.27**	-.35**	1	-.35**	.37**	.31**	.14
4	Agreement	.25*	-.20*	-.19	1	-.76**	-.55**	-.70**
5	Falsity	-.29**	-.16	.22*	-.69**	1	.49**	.62**
6	Manipulation	-.17	-.16	.25*	-.12	.16	1	.57**
7	Offensiveness	-.14	-.05	.07	-.58**	.58**	.15	1

Note. Results below the diagonal pertain to the IVN condition, while results above pertain to the WCVN condition.

Assumption Checks and Robustness Analyses

Assumptions of the Analysis of Variance.

To evaluate whether the one-way between-subjects ANOVAs met their underlying assumptions, we examined independence of observations, homogeneity of variance, and normality. Independence was ensured by the study design because participants were randomly assigned to one narrative condition and each participant contributed only one observation to each analysis. Homogeneity of variance was assessed using both the conventional mean-centered Levene test and the median-centered Brown–Forsythe variant. The latter is less sensitive to non-normality and extreme observations and was included as a complementary check. We also report the variance ratio, defined as the larger group variance divided by the smaller, to indicate the magnitude rather than merely the statistical detectability of heterogeneity. Variance ratios below approximately 1.5 are unlikely to substantially affect the conventional F test in balanced designs (Blanca et al., 2018). Normality was assessed within each condition using the Shapiro–Wilk test, skewness, and excess kurtosis.

The Levene and Brown–Forsythe tests indicated unequal variances for eight of the nine outcome variables; comprehensibility was the only exception (see Table S4). Normality was also violated in most condition-by-

variable cells (See Table S5). We therefore conducted Welch tests (Welch, 1951) and Yuen 20% trimmed-mean tests (Yuen, 1974) as sensitivity analyses.

Table S4. *Tests of Homogeneity of Variance for Outcome Variables in Study 1*

**Outcome Variable**	**Variance ratio**	**Levene's *F***	***p***	**Brown–Forsythe *F***	***p***
Discrimination against Men	2.69	21.61	< .001	18.87	< .001
Discrimination against Women	1.65	7.81	.006	6.20	.014
Gender Role Legitimacy	1.43	4.96	.027	5.03	.026
Agreement	2.26	25.79	< .001	28.48	< .001
Falsity	1.98	17.01	< .001	18.39	< .001
Manipulation	2.48	21.86	< .001	15.60	< .001
Offensiveness	1.99	13.80	< .001	9.17	.003
Comprehensibility	1.78	1.18	.278	2.33	.128
Effortfulness	1.68	16.60	< .001	15.87	< .001

Note. Levene's test is centered on the mean; the Brown–Forsythe variant is centered on the median.

Table S5. *Tests of Normality for Outcome Variables in Study 1*

Outcome Variable	Skew	Kurtosis	*W*	*p*	Skew	Kurtosis	*W*	*p*
	**IVN (*n* = 99)**				**WCVN (*n* = 101)**			
Discrimination against Men	−1.49	3.48	.885	< .001	0.64	−0.29	.948	< .001
Discrimination against Women	−0.94	1.04	.936	< .001	−0.64	−0.34	.945	< .001
Gender Role Legitimacy	1.05	1.23	.920	< .001	0.29	−0.34	.983	.221
Agreement	−1.69	4.64	.753	< .001	−0.54	−0.61	.901	< .001
Falsity	1.53	3.29	.782	< .001	0.73	−0.21	.891	< .001
Manipulation	0.36	−0.52	.835	< .001	0.68	0.17	.896	< .001
Offensiveness	0.65	0.30	.858	< .001	0.78	0.14	.884	< .001
Comprehensibility	−0.21	−0.88	.798	< .001	−1.31	3.28	.808	< .001
Effortfulness	−0.21	0.16	.903	< .001	−0.10	−1.01	.920	< .001

Note. *W* = Shapiro–Wilk statistic. Skewness and kurtosis are bias-corrected sample estimates; kurtosis is reported as excess kurtosis, for which the normal-distribution reference value is zero.

Robust Alternatives to the Conventional F Test.

Because the assumptions of homogeneity of variance and normality were frequently violated, we conducted two complementary sensitivity analyses for all Study 1 outcomes. Welch's F test was used to examine mean differences without assuming equal variances. Yuen's test compared 20% trimmed means and was used to assess whether the conclusions were sensitive to unequal variances, non-normality, and observations in the tails of the distributions.

The Welch tests reproduced the results of the conventional ANOVAs for all nine outcomes. The IVN condition showed higher perceived discrimination against men, Welch's $F(1, 165.81) = 159.06$, $p < .001$, and lower gender-role legitimacy, Welch's $F(1, 193.25) = 13.02$, $p < .001$, than the WCVN condition. The difference in perceived discrimination against women remained nonsignificant, Welch's $F(1, 188.46) = 1.75$, $p = .19$. The

differences in agreement, perceived falsity, perceived manipulation, and effortfulness also remained significant, *ps* $\leq$ .003, whereas the difference in comprehensibility remained nonsignificant, $p = .29$.

The Yuen tests produced the same substantive conclusions for the primary outcomes. The IVN condition had a higher trimmed mean for perceived discrimination against men than the WCVN condition, $t(89.0) = 14.25$, $p < .001$, and a lower trimmed mean for gender-role legitimacy, $t(108.0) = -4.16$, $p < .001$. Perceived discrimination against women remained nonsignificant, $t(102.4) = 0.97$, $p = .334$. The differences in agreement, perceived falsity, perceived manipulation, and effortfulness also remained significant, *ps* $\leq$ .02, whereas comprehensibility remained nonsignificant, $p = .74$.

Offensiveness was the only outcome for which the conclusions differed across estimators. The difference was significant in both the conventional ANOVA, $F(1, 198) = 5.56$, $p = .02$, and the Welch test, $F(1, 180.43) = 5.60$, $p = .02$, but not in the Yuen test, $t(118.8) = -1.50$, $p = .14$. The trimmed means were 2.11 in the IVN condition and 2.38 in the WCVN condition. This discrepancy indicates that the difference in ordinary means was concentrated in the upper tail rather than reflecting a broad shift in the distributions. The distribution of offensiveness ratings is examined further in Table S7.

Taken together, the sensitivity analyses indicate that the primary findings concerning perceived discrimination and gender-role legitimacy were robust to unequal variances, departures from normality, and the use of ordinary versus trimmed means. Full results are reported in Table S6.

Table S6. *Conventional, Welch, and Yuen's 20% Trimmed-Mean Tests of the Narrative Effect in Study 1*

**Outcome Variable**	***F*(1, 198)**	***p***	**Welch's *F***	***df***	***p***
Discrimination against Men	157.60	< .001	159.06	1, 165.81	< .001
Discrimination against Women	1.74	.189	1.75	1, 188.46	.188
Gender Role Legitimacy	12.98	< .001	13.02	1, 193.25	< .001
Agreement	25.11	< .001	25.30	1, 174.44	< .001
Falsity	20.30	< .001	20.43	1, 180.72	< .001
Manipulation	19.24	< .001	19.40	1, 169.91	< .001
Offensiveness	5.56	.019	5.60	1, 180.43	.019
Comprehensibility	1.12	.292	1.12	1, 185.33	.291
Effortfulness	9.29	.003	9.33	1, 187.60	.003

Note. All conclusions converge across the three tests except for offensiveness, which is significant when means are compared and non-significant when the tails are trimmed. That difference lies in the upper tail of the distribution; Table S7 reports its location.

Table S6 (continued).

**Outcome Variable**	**Yuen's *t***	***df***	***p***	**Trimmed *M* IVN**	**Trimmed *M* WCVN**
Discrimination against Men	14.25	89.0	< .001	4.80	2.80
Discrimination against Women	0.97	102.4	.334	4.47	4.32
Gender Role Legitimacy	−4.16	108.0	< .001	2.49	2.96
Agreement	4.16	110.8	< .001	5.02	4.28
Falsity	−3.98	115.9	< .001	1.92	2.64
Manipulation	−4.16	106.9	< .001	1.89	2.49
Offensiveness	−1.50	118.8	.137	2.11	2.38
Comprehensibility	0.34	100.9	.735	5.21	5.16
Effortfulness	2.33	100.0	.022	3.87	3.34

Note. All conclusions converge across the three tests except for offensiveness, which is significant when means are compared and non-significant when the tails are trimmed. That difference lies in the upper tail of the distribution; Table S7 reports its location.

Distribution of Offensiveness Ratings.

The two conditions had identical median offensiveness ratings, indicating that most participants responded similarly to the two narratives. However, the difference between conditions was concentrated in the upper tail of the distribution. As an exploratory description, ratings of 5 or 6 were classified as strong offensiveness. Strong offensiveness was more frequent in the WCVN condition (9.9%) than in the IVN condition (1.0%). This pattern suggests that, although the two narratives elicited similar reactions among most men, the WCVN was more likely to provoke strong offensiveness among a small subset of participants. See Table S7 for details.

Table S7. *Distribution of Offensiveness Ratings by Narrative Condition in Study 1*

**Rating**	**WCVN *n* (%)**	**IVN *n* (%)**
1.00	22 (21.8)	22 (22.2)
2.00	34 (33.7)	48 (48.5)
3.00	27 (26.7)	21 (21.2)
4.00	8 (7.9)	7 (7.1)
5.00	8 (7.9)	1 (1.0)
6.00	2 (2.0)	0 (0.0)
Exploratory threshold	WCVN n (%)	IVN n (%)
≥ 5	10 (9.9)	1 (1.0)

Note. Ratings were made on a six-point scale. The comparison of ratings of 5 or 6 was an exploratory upper-tail analysis. Fisher's exact test indicated that strong offensiveness was more frequent in the WCVN condition than in the IVN condition, $p = .01$, OR = 10.77. WCVN $n = 101$; IVN $n = 99$.

One-way Between-subject ANCOVA

A one-way between-subjects ANCOVA was conducted to compare the effect of narrative (the WCVN vs. the IVN), after controlling for participants' sociodemographic characteristics, which were age, education, monthly income, and subjective socioeconomic status. The results indicated a significant effect of narrative on discrimination against men ($F(1, 194) = 152.69, p < .001, \eta^2_p = .44$), gender role legitimacy ($F(1, 194) = 11.29, p < .001, \eta^2_p = .06$), agreement ($F(1, 194) = 24.50, p < .001, \eta^2_p = .11$), falsity ($F(1, 194) = 19.78, p < .001, \eta^2_p = .09$), manipulation ($F(1, 194) = 21.04, p < .001, \eta^2_p = .10$), and offensiveness ($F(1, 194) = 5.90, p = .02, \eta^2_p = .03$), but not on discrimination against women ($F(1, 194) = 1.33, p = .25, \eta^2_p = .01$). The results were consistent with the unadjusted ANOVA results reported in main text. See further illustration in Table S8.

To examine whether the Study 1 findings could be explained by differences in perceived reading effort,

a series of one-way between-subjects ANCOVAs was conducted with narrative condition (the WCVN vs. the IVN) as the independent variable and effortfulness as a covariate. The effect of narrative remained significant for discrimination against men ($F(1, 197) = 146.34$, $p < .001$, $\eta^2_p = .43$), gender role legitimacy ($F(1, 197) = 13.35$, $p < .001$, $\eta^2_p = .06$), agreement ($F(1, 197) = 24.74$, $p < .001$, $\eta^2_p = .11$), falsity ($F(1, 197) = 19.67$, $p < .001$, $\eta^2_p = .09$), manipulation ($F(1, 197) = 20.15$, $p < .001$, $\eta^2_p = .09$), and offensiveness ($F(1, 197) = 6.30$, $p = .01$, $\eta^2_p = .03$), but not for discrimination against women ($F(1, 197) = 0.97$, $p = .33$, $\eta^2_p = .01$). Effortfulness was not significantly associated with any outcome, *p*s = .15–.84. Thus, the main findings remained unchanged after controlling for perceived reading effort. See Table S8.

Table S8. *One-way ANCOVA Analyses of Narrative Effect on Outcome Variables in Study 1*

Outcome Variable	*Control for Sociodemographics*			*Control for Effortfulness*		
	F	*p*	η_p^2	*F*	*p*	η_p^2
Discrimination against Men	152.69	< .001	.44	146.34	< .001	.43
Discrimination against Women	1.33	.25	.01	0.97	.33	.01
Gender Role Legitimacy	11.29	< .001	.06	13.35	< .001	.06
Agreement	24.50	< .001	.11	24.74	< .001	.11
Falsity	19.78	< .001	.09	19.67	< .001	.09
Manipulation	21.04	< .001	.10	20.15	< .001	.09
Offensiveness	5.90	.02	.03	6.30	.01	.03

Exploratory Moderation Analyses of Ideological Variables

To examine whether the effects of narrative condition varied across ideological orientations, we conducted a series of exploratory moderation analyses using PROCESS v4.2 for SPSS, Model 1. Hostile sexism and Identification with Masculinity were separately tested as moderators of the effect of narrative condition on perceived discrimination against men, perceived discrimination against women, and gender role legitimacy.

Perceived discrimination against men.

Hostile sexism significantly moderated the effect of narrative condition on perceived discrimination against men, b = -0.593, SE = 0.129, t = -4.61, p < .001, ΔR^2 = .044. Conditional effects indicated that the IVN significantly increased perceived discrimination against men at low, mean, and high levels of hostile sexism, low: b = 2.250, SE = 0.170, t = 13.22, p < .001; mean: b = 1.693, SE = 0.118, t = 14.29, p < .001; high: b = 1.135, SE = 0.168, t = 6.74, p < .001. Johnson–Neyman analysis showed that the effect of narrative remained significant until hostile sexism reached approximately 1.94 SD above the mean; beyond this point, the conditional effect was no longer statistically significant.

Identification with Masculinity did not significantly moderate the effect of narrative condition, b = -0.138, SE = 0.169, t = -0.82, p = .416, ΔR^2 = .002.

Taken together, these results indicate that the IVN robustly increased perceived discrimination against men. When moderation emerged, it reflected a weaker IVN advantage among men higher in hostile sexism; the effect was not qualified by identification with masculinity.

Perceived discrimination against women.

Neither hostile sexism nor identification with masculinity significantly moderated the effect of narrative condition on perceived discrimination against women. The interaction terms were nonsignificant for hostile sexism, b = 0.217, SE = 0.153, t = 1.42, p = .158, ΔR^2 = .01, and Identification with Masculinity, b = 0.006, SE = 0.175, t = 0.03, p = .974, ΔR^2 < .001.

Gender role legitimacy.

For gender role legitimacy, hostile sexism showed a marginal interaction with narrative condition, b = -0.240, SE = 0.125, t = -1.92, p = .056, ΔR^2 = .017. Conditional effect analyses indicated that the IVN did not significantly reduce gender role legitimacy among men low in hostile sexism, b = -0.209, SE = 0.165, t = -1.27, p

= .207, but did significantly reduce gender role legitimacy among men at the mean level of hostile sexism, b = -0.435, SE = 0.115, t = -3.78, p < .001, and at higher levels of hostile sexism, b = -0.661, SE = 0.164, t = -4.04, p < .001. Johnson–Neyman analysis indicated that the effect of narrative became significant when hostile sexism was approximately 0.65 SD below the mean or higher. Estimated values from the hostile sexism model suggested that men low in hostile sexism already reported relatively low gender role legitimacy across conditions (M_{WCVN} = 2.79, M_{IVN} = 2.58), whereas the IVN advantage was more pronounced at the mean level (M_{WCVN} = 3.02, M_{IVN} = 2.59), and high levels of hostile sexism (M_{WCVN} = 3.26, M_{IVN} = 2.60).

Identification with Masculinity did not significantly moderate the effect of narrative condition on gender role legitimacy, b = 0.011, SE = 0.140, t = 0.08, p = .939, ΔR^2 < .001.

Overall, hostile sexism showed some evidence of moderating the effect of narrative condition, whereas identification with masculinity did not. The IVN increased men's perceived discrimination against men broadly, but its effect on gender role legitimacy appeared more pronounced among men with relatively higher hostile sexism.

Supplementary Analyses in Study 2

Descriptive Statistics for the 3 × 2 × 2 Design.

Table S9 reports the sample size, mean, standard deviation, standard error, and 95% confidence interval for all 12 Narrative × Target × Content cells plotted in Figure 1 of the main text. Each narrative condition included 90 participants, and there were no missing observations across the four repeated measures.

Table S9. *Means, Standard Deviations, and 95% Confidence Intervals for All 12 Narrative × Target × Content Cells in Study 2*

**Target**	**Content**	**Narrative**	***n***	***M***	***SD***	***SE***	**95% CI**
Men	Masculine	Control	90	5.04	0.51	.054	[4.93, 5.14]
Men	Masculine	WCVN	90	4.99	0.54	.057	[4.87, 5.10]
Men	Masculine	IVN	90	4.81	0.73	.076	[4.66, 4.96]
Men	Feminine	Control	90	4.23	0.62	.066	[4.10, 4.36]
Men	Feminine	WCVN	90	4.27	0.68	.072	[4.12, 4.41]
Men	Feminine	IVN	90	3.96	0.76	.080	[3.80, 4.12]
Women	Masculine	Control	90	4.01	0.71	.075	[3.86, 4.15]
Women	Masculine	WCVN	90	4.10	0.72	.076	[3.94, 4.25]
Women	Masculine	IVN	90	3.74	0.76	.080	[3.58, 3.90]
Women	Feminine	Control	90	4.90	0.57	.060	[4.78, 5.01]
Women	Feminine	WCVN	90	4.81	0.71	.075	[4.66, 4.96]
Women	Feminine	IVN	90	4.81	0.72	.076	[4.66, 4.96]

Note. CI = confidence interval. Confidence intervals are computed within cell.

Assumption Checks and Robustness Analyses

Assumptions of the Mixed-Design Analysis of Variance.

To evaluate whether the 3 × 2 × 2 mixed-design ANOVA met its underlying assumptions, we examined independence of observations, homogeneity of covariance matrices, sphericity, homogeneity of variance, and normality. Independence between participants was ensured by random assignment to one of the three narrative conditions, and all 270 participants provided complete responses for the four repeated measures. Dependence among each participant's repeated ratings was modeled by the within-subjects factors. Homogeneity of covariance matrices was assessed using Box's *M*. Because Target and Content each had only two levels, sphericity was satisfied by definition and no correction was required.

Homogeneity of variance and normality were examined at the four error strata used by the mixed model: the participant-level mean and the Target, Content, and Target × Content contrasts. At each stratum, homogeneity of variance was assessed using both the conventional mean-centered Levene test and the median-centered Brown–Forsythe variant, together with variance ratios. The four raw Target × Content cells were also examined because the exploratory follow-up analyses compared narrative conditions separately within each cell. Normality was assessed using the Shapiro–Wilk test, skewness, and excess kurtosis for the same four error strata.

Box's test was not statistically significant, Box's $M = 29.17$, $F(20, 255{,}895.98) = 1.43$, $p = .10$. Neither the Levene nor the Brown–Forsythe tests were significant at any of the four model error strata (Levene *p*s = .31–.96; Brown–Forsythe *p*s = .41–.95). At the raw-cell level, only prescriptions that men should display masculine traits showed clear evidence of unequal variances across narrative conditions, Levene's $F(2, 267) = 4.11$, $p = .018$, variance ratio = 2.03; the corresponding Brown–Forsythe test was marginal, $F(2, 267) = 3.00$, $p = .05$. Normality tests detected departures at several strata, with the most pronounced leptokurtosis at the between-subjects and Target-contrast strata (excess kurtosis = 2.23 and 2.18, respectively). We therefore retained the mixed ANOVA

as the primary analysis and conducted two complementary sensitivity analyses: Welch and Games–Howell analyses for the overall Narrative effect and the exploratory cell-specific effects, and randomization-based analyses for all seven mixed-design effects. Full assumption checks are summarized in Table S10 and reported in detail in Tables S11 and S12.

Table S10. *Assumptions of the Mixed-Design Analysis of Variance in Study 2*

Assumption	Where it applies	How assessed	Outcome
1. Independence of participants	Between-subjects factor	Design: random assignment to narrative condition; dependence among each participant's four repeated measures is modelled by the design	Ensured by design
2. Homogeneity of covariance matrices	Full covariance structure across the three conditions	Box's *M*	Not significant, Box's $M = 29.17$, $F(20, 255{,}895.98) = 1.43$, $p = .097$
3. Sphericity	Within-subjects factors	Not applicable: each within-subjects factor has two levels	Satisfied by definition; no correction required
4. Homogeneity of variance	The four error strata of the model; also examined for each raw cell	Levene's test and Brown–Forsythe test (Panels B and C)	Not significant at any of the four strata; significant in one raw cell (Men / Masculine)
5. Normality	The same four error strata	Shapiro–Wilk test, skewness, kurtosis (tables below)	Mild leptokurtosis at the between-subjects and Target-contrast strata; Table S15 re-tests every effect in a randomization-based sensitivity analysis

Table S11. *Tests of Homogeneity of the Model Error Strata for Study 2*

Panel A.<br>Error strata of the mixed model	Variance ratio	Levene's F(2, 267)	p	Brown–Forsythe F(2, 267)	p
Between-subjects stratum (subject means)	1.79	1.04	.357	0.90	.408
Within-subjects stratum: Target contrast	1.18	0.14	.867	0.10	.909
Within-subjects stratum: Content contrast	1.23	0.04	.964	0.05	.952
Within-subjects stratum: Target × Content contrast	1.30	1.19	.306	0.67	.513
Panel B.<br>Raw cell scores	Variance ratio	Levene's F(2, 267)	p	Brown–Forsythe F(2, 267)	p
Men / Masculine	2.03	4.11	.018	3.00	.051
Men / Feminine	1.49	0.93	.396	0.73	.484
Women / Masculine	1.15	0.23	.795	0.25	.782
Women / Feminine	1.61	2.06	.129	1.62	.199

Note. Variance ratios are the larger group variance divided by the smaller, across the three narrative conditions (n = 90 each). Panel A entries are the variables against which the mixed model's F tests are computed: the subject mean for the between-subjects effect, and the corresponding within-subject contrast for each within-subjects effect and its interaction with narrative condition.

Table S12. *Tests of Normality of the Model Error Strata for Study 2*

Error stratum	Control		WCVN		IVN		Pooled residuals *W*	*p*	Skew	Kurtosis
	W	*p*	*W*	*p*	*W*	*p*				
Between-subjects stratum (subject means)	.979	0.156	.972	0.053	.937	<.001	.966	<.001	−0.79	2.23
Within-subjects stratum: Target contrast	.921	<.001	.949	0.001	.958	0.005	.950	<.001	0.94	2.18
Within-subjects stratum: Content contrast	.986	0.479	.968	0.028	.946	<.001	.990	0.068	−0.07	0.90
Within-subjects stratum: Target × Content contrast	.980	0.173	.949	0.001	.972	0.049	.974	<.001	0.51	−0.17

Note. W = Shapiro–Wilk statistic. Pooled residuals are the stratum scores centered within narrative condition (n = 270). Skewness and kurtosis are bias-corrected sample estimates; kurtosis is reported as excess kurtosis, for which the normal-distribution reference value is zero.

Robust Alternatives to the Conventional F Test.

Because the Men/Masculine cell showed unequal variances across narrative conditions, we conducted Welch's omnibus tests and Games–Howell pairwise comparisons as sensitivity analyses. Welch's test examines the same equality-of-means hypothesis as the conventional ANOVA while allowing group variances to differ. Games–Howell comparisons likewise do not assume equal variances and control the familywise error rate across the three pairwise comparisons within each analysis (Games & Howell, 1976; Hancock & Klockars, 1996). For consistency, these analyses were conducted for the overall Narrative effect and for each of the four Target × Content cells.

The four cell-specific analyses were exploratory because the Narrative × Target × Content interaction was not statistically significant. They were therefore used to describe where the overall Narrative pattern appeared

rather than to establish that the effects differed reliably across the four cells. No additional correction was applied across the overall analysis and the four exploratory cell-specific analyses.

The overall Narrative effect remained significant under Welch's test, $F(2, 175.57) = 4.51$, $p = .01$. Games–Howell comparisons showed lower overall stereotype endorsement in the IVN condition than in the control condition, mean difference = 0.21, 95% CI [0.03, 0.39], $p = .016$, Hedges' $g = 0.41$, and the WCVN condition, mean difference = 0.21, 95% CI [0.02, 0.40], $p = .028$, Hedges' $g = 0.39$. The control and WCVN conditions did not differ, $p = .999$.

For prescriptions that men should display feminine traits, the Narrative effect remained significant under Welch's test, $F(2, 176.86) = 4.76$, $p = .01$. Games–Howell comparisons showed lower endorsement in the IVN condition than in both the control and WCVN conditions, *ps* = .03 and .01, respectively. The same pattern was obtained for prescriptions that women should display masculine traits, Welch's $F(2, 177.85) = 5.50$, $p = .005$; endorsement was lower in the IVN condition than in the control and WCVN conditions, *ps* = .04 and .004, respectively. By contrast, the Narrative effect on prescriptions that women should display feminine traits remained nonsignificant, Welch's $F(2, 175.77) = 0.58$, $p = .56$.

The result for prescriptions that men should display masculine traits was less robust. The conventional ANOVA was significant, $F(2, 267) = 3.51$, $p = .03$, whereas the Welch omnibus test did not reach statistical significance, $F(2, 174.72) = 2.97$, $p = .054$. The Games–Howell comparison nevertheless indicated lower endorsement in the IVN condition than in the control condition, $p = .04$, whereas the IVN and WCVN conditions did not differ, $p = .162$.

Taken together, the overall Narrative effect was robust across the conventional and unequal-variance analyses. The exploratory cell-specific pattern was also generally reproduced, although the evidence concerning

prescriptions that men should display masculine traits was weaker. The conventional and Welch omnibus tests are reported in Table S13, and the Games–Howell pairwise comparisons are reported in Table S14.

Table S13. Conventional and Welch Omnibus Tests of Narrative Effects Overall and Within Each Cell in Study 2

Panel A. Omnibus tests	*F*(2, 267)	*p*	Welch's *F*	*df*	*p*
Overall (Narrative main effect)	5.17	.006	4.51	2, 175.57	.012
Men / Masculine	3.51	.031	2.97	2, 174.72	.054
Men / Feminine	5.33	.005	4.76	2, 176.86	.010
Women / Masculine	5.75	.004	5.50	2, 177.85	.005
Women / Feminine	0.50	.605	0.58	2, 175.77	.560

Note. "Overall" refers to the between-subjects Narrative effect calculated from each participant's mean across the four Target × Content cells.

Table S14. Games–Howell Pairwise Comparisons of Narrative Conditions Overall and Within Each Cell in Study 2

	Comparison	Mean difference	95% CI	*df*	*p*	Hedges' *g*
Overall	Control vs. WCVN	0.00	[−0.16, 0.17]	174.28	.999	0.01
	Control vs. IVN	0.21	[0.03, 0.39]	164.80	.016	0.41
	WCVN vs. IVN	0.21	[0.02, 0.40]	174.44	.028	0.39
Men / Masculine	Control vs. WCVN	0.05	[−0.14, 0.24]	177.23	.796	0.10
	Control vs. IVN	0.23	[0.01, 0.45]	159.50	.044	0.36
	WCVN vs. IVN	0.17	[−0.05, 0.40]	164.96	.162	0.27
Men / Feminine	Control vs. WCVN	−0.04	[−0.27, 0.19]	176.69	.918	−0.06
	Control vs. IVN	0.27	[0.03, 0.52]	171.36	.027	0.39
	WCVN vs. IVN	0.31	[0.05, 0.56]	175.76	.013	0.42
Women / Masculine	Control vs. WCVN	−0.09	[−0.34, 0.16]	177.94	.684	−0.12
	Control vs. IVN	0.27	[0.01, 0.53]	177.13	.042	0.36
	WCVN vs. IVN	0.36	[0.09, 0.62]	177.52	.004	0.48
Women / Feminine	Control vs. WCVN	0.09	[−0.14, 0.31]	170.51	.632	0.14
	Control vs. IVN	0.09	[−0.14, 0.32]	168.87	.649	0.13
	WCVN vs. IVN	0.00	[−0.25, 0.25]	177.90	1.000	0.00

Note. Mean differences are calculated as the first condition minus the second condition. Games–Howell *p* values and 95% confidence intervals are adjusted across the three pairwise comparisons within each analysis using the studentized range distribution. Hedges' *g* was calculated using the pooled standard deviation with the small-sample correction.

Randomization-Based Sensitivity Analyses of the Mixed-Design Effects.

To assess whether departures from normality at the model error strata affected the mixed-design results, all seven effects were re-tested using 10,000 randomizations. Effects involving Narrative were tested by permuting the narrative-condition labels while preserving the three group sizes of $n = 90$. The within-subjects main effects were tested by sign flipping each participant's contrast score. Monte Carlo p values were right-tailed and calculated as $(b + 1)/(B + 1)$, using the NumPy PCG64 generator with seed 20260726.

The randomization-based analyses reproduced the inferential pattern of the parametric mixed ANOVA. The main effect of Narrative remained significant, $p = .006$. The Narrative × Target interaction, $p = .39$, the Narrative × Content interaction, $p = .14$, and the Narrative × Target × Content interaction, $p = .07$, remained nonsignificant, providing no clear evidence that the effect of Narrative varied according to target gender or stereotype content, either separately or jointly. The Target, Content, and Target × Content effects also retained the same significance decisions, randomization *p*s < .001, .03, and < .001, respectively. Every randomization *p* value differed from its parametric counterpart by no more than .006. Full results are reported in Table S15.

Table S15. *Randomization-Based Sensitivity Analyses of the Study 2 Mixed Design*

**Effect**	***F***	***df***	**Parametric *p***	**Randomization *p***	***b***	**Procedure**
Narrative	5.17	2, 267	.006	.006	55	Permutation of narrative labels
Target	43.84	1, 267	< .001	< .001	0	Sign flipping of contrast scores
Content	4.45	1, 267	.036	.030	302	Sign flipping of contrast scores
Target × Content	382.61	1, 267	< .001	< .001	0	Sign flipping of contrast scores
Narrative × Target	0.95	2, 267	.390	.387	3869	Permutation of narrative labels
Narrative × Content	1.98	2, 267	.140	.138	1379	Permutation of narrative labels
Narrative × Target × Content	2.71	2, 267	.068	.070	695	Permutation of narrative labels

Note. b = number of replicates whose F equalled or exceeded the observed F; B = 10,000 for every test; $p = (b + 1)/(B + 1)$, so the smallest attainable value is 1/(10,000 + 1) = .00010. Analyses used the NumPy PCG64 generator with seed 20260726.

Three-way Mixed-design ANCOVA

A 3(Narrative) × 2(Target) × 2(Content) mixed-design ANCOVA was conducted, with participants' reading experiences and sociodemographic characteristics as covariates. Significant main effects were obtained for Narrative ($F(2, 260) = 5.68$, $p = .004$, $\eta^2_p = .04$; $M_{IVN} = 4.32$, $SE = 0.05$; $M_{WCVN} = 4.54$, $SE = 0.05$; $M_{control} = 4.54$, $SE = 0.05$) and marginally for Target ($F(1, 260) = 3.08$, $p = .08$, $\eta^2_p = .01$; $M_{men} = 4.55$, $SE = 0.03$; $M_{women} = 4.39$, $SE = 0.03$). However, the main effect of Content ($p = .13$) and the interactions, including both two-way and three-way interactions, were non-significant (ps ≥ .10). Pairwise comparisons indicated that men who read the IVN were less likely to endorse prescriptive gender stereotypes in general compared to the WCVN ($p = .01$) and control conditions ($p = .01$), whereas the difference between the WCVN condition and the control condition was negligible ($p = 1.00$).

The three-way interaction was not significant ($F(2, 260) = 2.30$, $p = .10$, $\eta^2_p = .02$). To provide further information about the pattern across specific role prescriptions, we conducted exploratory follow-up analyses of the simple effect of Narrative within each Target × Content cell. Because the three-way interaction was not significant, these analyses should not be interpreted as evidence that the Narrative effect differed reliably across the four cells. Pairwise comparisons were Bonferroni-adjusted. A significant main effect of Narrative was observed when men were instructed to indicate whether women should display masculine personalities ($F(2, 260) = 5.67$, $p = .004$, $\eta^2_p = .04$); whether men should display masculine personalities ($F(2, 260) = 3.86$, $p = .02$, $\eta^2_p = .03$); and whether men should display feminine personalities ($F(2, 260) = 5.45$, $p = .005$, $\eta^2_p = .04$). Similar patterns emerged across these conditions, with participants in the IVN condition reporting significantly lower endorsement compared to those in the WCVN and control conditions (ps < .04). Furthermore, no significant differences were observed between the WCVN and control conditions (ps = 1.00). In contrast, when men were asked to indicate whether women should display feminine traits, the effect of Narrative was not significant (F(2,

260) = .58, p = .56, η^2_p = .004). Participants across the three narrative conditions demonstrated similar levels of endorsement (ps > .89). Figure S1 presents a visual representation of the results. The main findings of ANCOVA were consistent with those of ANOVA reported in the main article.

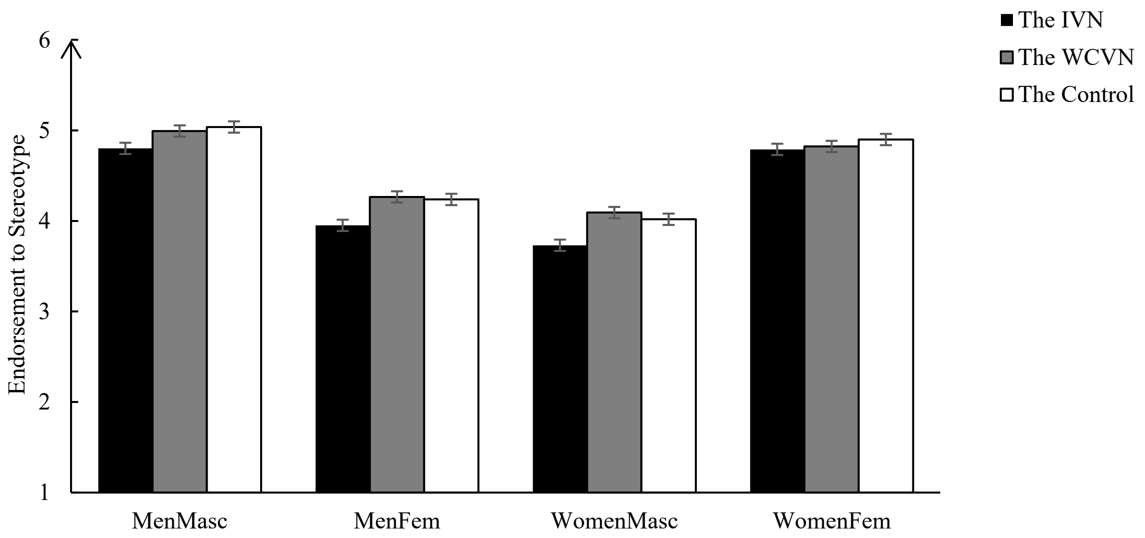

Figure S1. Mean Stereotype Endorsement as a Function of Target, Content, and Narrative.
Note. Error bars represent standard errors.

References

Blanca, M. J., Alarcón, R., Arnau, J., Bono, R., & Bendayan, R. (2018). Effect of variance ratio on ANOVA robustness: Might 1.5 be the limit? *Behavior Research Methods*, *50*(3), 937–962. https://doi.org/10.3758/s13428-017-0918-2

Games, P. A., & Howell, J. F. (1976). Pairwise multiple comparison procedures with unequal *n*'s and/or variances: A Monte Carlo study. *Journal of Educational Statistics*, *1*(2), 113–125. https://doi.org/10.3102/10769986001002113

Hancock, G. R., & Klockars, A. J. (1996). The quest for α: Developments in multiple comparison procedures in the quarter century since Games (1971). *Review of Educational Research*, *66*(3), 269–306. https://doi.org/10.3102/00346543066003269

Welch, B. L. (1951). On the comparison of several mean values: An alternative approach. *Biometrika*, *38*(3–4), 330–336. https://doi.org/10.1093/biomet/38.3-4.330

Yuen, K. K. (1974). The two-sample trimmed *t* for unequal population variances. *Biometrika*, *61*(1), 165–170. https://doi.org/10.1093/biomet/61.1.165
